# Interaction between HLA-G and NK cell receptor KIR2DL4 orchestrates HER2-positive breast cancer resistance to trastuzumab

**DOI:** 10.1038/s41392-021-00629-w

**Published:** 2021-06-23

**Authors:** Guoxu Zheng, Zhangyan Guo, Weimiao Li, Wenjin Xi, Baile Zuo, Rui Zhang, Weihong Wen, An-Gang Yang, Lintao Jia

**Affiliations:** 1 State Key Laboratory of Cancer Biology, Department of Immunology, Fourth Military Medical University, Xi’an, China; 2 Department of Oncology, The Second Affiliated Hospital of Xi’an Jiaotong University, Xi’an, China; 3 State Key Laboratory of Cancer Biology, Department of Biochemistry and Molecular Biology, Fourth Military Medical University, Xi’an, China

**Keywords:** Breast cancer, Tumour immunology

## Abstract

Despite the successful use of the humanized monoclonal antibody trastuzumab (Herceptin) in the clinical treatment of human epidermal growth factor receptor 2 (HER2)-overexpressing breast cancer, the frequently occurring drug resistance remains to be overcome. The regulatory mechanisms of trastuzumab-elicited immune response in the tumor microenvironment remain largely uncharacterized. Here, we found that the nonclassical histocompatibility antigen HLA-G desensitizes breast cancer cells to trastuzumab by binding to the natural killer (NK) cell receptor KIR2DL4. Unless engaged by HLA-G, KIR2DL4 promotes antibody-dependent cell-mediated cytotoxicity and forms a regulatory circuit with the interferon-γ (IFN-γ) production pathway, in which IFN-γ upregulates KIR2DL4 via JAK2/STAT1 signaling, and then KIR2DL4 synergizes with the Fcγ receptor to increase IFN-γ secretion by NK cells. Trastuzumab treatment of neoplastic and NK cells leads to aberrant cytokine production characterized by excessive tumor growth factor-β (TGF-β) and IFN-γ, which subsequently reinforce HLA-G/KIR2DL4 signaling. In addition, TGF-β and IFN-γ impair the cytotoxicity of NK cells by upregulating PD-L1 on tumor cells and PD-1 on NK cells. Blockade of HLA-G/KIR2DL4 signaling improved the vulnerability of HER2-positive breast cancer to trastuzumab treatment in vivo. These findings provide novel insights into the mechanisms underlying trastuzumab resistance and demonstrate the applicability of combined HLA-G and PD-L1/PD-1 targeting in the treatment of trastuzumab-resistant breast cancer.

## Introduction

Breast cancers overexpressing human epidermal growth factor receptor 2 (HER2) are characterized by high metastatic potential and poor patient prognosis.^1^ The humanized monoclonal antibody trastuzumab, also known as Herceptin, has been successfully used for the treatment of HER2-positive metastatic breast cancer or early-stage neoplasms that have spread to the lymph nodes or exhibit other high-risk features.^2^ Although trastuzumab alone, and in combination with chemotherapy, significantly prolong disease-free survival, the overall response rate to trastuzumab is not satisfactory, and patients initially responsive to trastuzumab usually develop resistance within a year.^2,3^ A recent clinical study has indicated that only 15.6% (53/339) of patients bearing HER2-positive metastatic breast cancer were long-term responders to treatment with trastuzumab and the chemotherapy drug docetaxel.^4^ The mechanisms underlying primary or acquired resistance to trastuzumab remain to be fully elucidated.

Accumulating evidence has shown that the immune system contributes substantially to the therapeutic efficacy of trastuzumab in breast cancer.^5^ Trastuzumab binds via its immunoglobulin G1 (IgG1) Fc portion to the Fcγ receptor on immune effector cells, mainly FcγRIII (CD16) on natural killer (NK) cells, and elicits the release of cytotoxic factors, a process known as antibody-dependent cell-mediated cytotoxicity (ADCC).^6^ Clinical studies revealed that polymorphisms in FcγR that play roles in ADCC can be used to predict the clinical outcome of HER2-positive breast cancer patients receiving trastuzumab treatment, suggesting that NK cells and ADCC are critically involved in the responsiveness of patients to trastuzumab.^7^ CD16 often associates with the FcεRI γ-chain (FcRγ) within the cell membrane.^8^ The binding of the IgG immune complex to CD16 causes phosphorylation of the immune tyrosine-based activation motif (ITAM) of FcRγ and the recruitment of tyrosine kinases ZAP-70 and Syk.^8^ Subsequently, downstream pathways, including the PI3K/Akt, NF-κB, and ERK pathways, are activated, leading to NK cell degranulation, cytokine secretion, and finally cell lysis.^8^

The activity of NK cells is tightly regulated by a balance of signaling from inhibitory and activating receptors.^9^ NK cells constitutively express germline-encoded inhibitory receptors that recognize major histocompatibility complex (MHC) class I molecules and provide inhibitory signals for self-tolerance.^10^ These inhibitory receptors in humans include killer cell Ig-like receptors (KIRs) and CD94-NKG2A heterodimers.^11^ KIR2DL4 is an atypical KIR family member that contains both an arginine–tyrosine activation motif in its transmembrane region and an immunoreceptor tyrosine-based inhibitory motif (ITIM) in its cytoplasmic tail, suggesting that KIR2DL4 may function as an activating or inhibitory receptor.^12^ Nonetheless, it is unknown whether KIR2DL4 is involved in trastuzumab-induced ADCC or how it is regulated in breast cancer. NK cells also express activating receptors such as CD16, NKG2D, NKp44, NKp46, and DNAM-1 (CD226).^9,13^ These receptors recognize stress ligands, pathogen-encoded ligands, and antibodies to trigger NK cell cytotoxicity, which is mediated by the release of perforin, granzymes, and Fas ligand.^14^ However, it remains largely unclear how these receptors regulate ADCC by coupling to the CD16 signaling pathway. In addition, NK cells are an essential source of cytokines, including interleukin-2 (IL-2), interferon-γ (IFN-γ), and chemokines.^15^ The intensity and quality of activation of NK cells depend on the cytokines present and their cross-talk with other types of cells in the tissue microenvironment.^16^

NK cells are key players in antitumor immunity.^17^ Previous studies have shown that the tumoricidal activity of NK cells is controlled by immunological checkpoint molecules such as NKG2A/CD94, TIGIT, and Tim-3.^9,18,19^ Tumor cells expressing high levels of HLA class I molecules can also inhibit NK cells through the engagement of KIRs.^20^ Thus, blocking the interaction between HLA molecules and inhibitory receptors is an important strategy to therapeutically enhance NK cell responses. HLA-G is a nonclassical MHC class I molecule consisting of globular domains (α1, α2, and α3), a transmembrane domain, and a cytoplasmic domain; HLA-G1 is the complete isoform associated with β2-microglobulin.^21^ Although originally identified as a mediator of immune tolerance in the maternal–placental interface, HLA-G is an immune checkpoint molecule with specific relevance in cancer immune escape.^22^ HLA-G is also the only known ligand of KIR2DL4 on NK cells, although the role of HLA-G/KIR2DL4 signaling in antitumor immunity is still uncharacterized.^23^ In this study, we identified HLA-G as a pivotal mediator of breast cancer resistance to trastuzumab, evaluated the role of microenvironmental cytokines in the regulation of trastuzumab-mediated ADCC, and examined the effect of blocking the HLA-G/KIR2DL4 interaction on the antitumor activity of NK cells.

## Results

### HLA-G expression predicts a low trastuzumab response in HER2-positive breast cancer

**Fig. 1:**
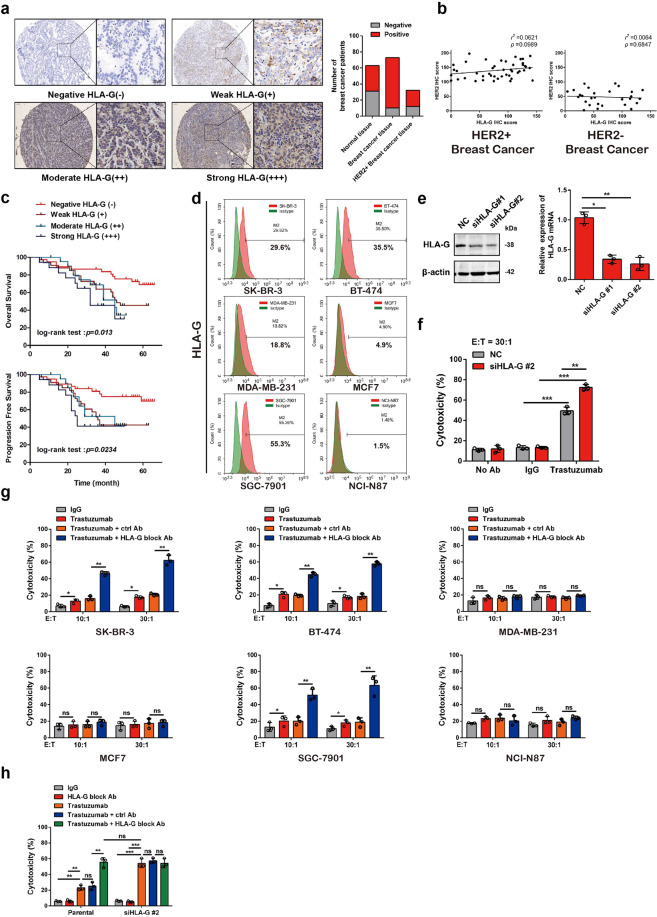
HLA-G depletion on HER2-positive breast cancer cells enhances trastuzumab-induced lysis by cocultured NK cells. **a** Representative immunohistochemical staining of HLA-G in a breast cancer tissue array. (Right panel) The number of HLA-G-positive and -negative normal and neoplastic tissues obtained from patients with HER2-positive or other subtypes of breast cancer were plotted. **b** Correlation analysis for HLA-G and HER2 expression in breast cancer tissues. **c** Kaplan–Meier survival analysis based on the prognosis and recurrence rates of 108 HER2-positive breast cancer patients grouped by HLA-G expression. **d** FCM examination of HLA-G expression in breast cancer and gastric cancer cell lines. **e** Western blot analysis (left) and qRT-PCR assay (right) of HLA-G expression in SK-BR-3 cells 48 h after transfection with the indicated siRNA. **f** NK cells prepared from PBMCs of healthy donors (effector, abbreviated as “E”) were cocultured with control or HLA-G siRNA-transfected SK-BR-3 cells (target, abbreviated as “T”) supplemented with or without the indicated antibodies. The cytotoxicity of the NK cells was measured via FCM as described in the “Materials and methods.” **g** NK cells in the presence of trastuzumab were cocultured with different neoplastic cell lines at the indicated E:T ratios supplemented with or without a control or HLA-G blocking antibody. The cytotoxicity of NK cells was measured via FCM. **h** Parental or HLA-G knockdown (siHLA-G#2) SK-BR-3 cells were cocultured with NK cells (E:T = 10:1) in the presence of trastuzumab with or without an HLA-G-blocking antibody. The cytotoxicity of NK cells was measured via FCM. All experiments were performed three times. Statistical significance was determined by Student’s *t* test. *P < 0.05, **P < 0.01, and ***P < 0.001. n.s. Nonsignificant

We first examined whether HLA-G, a nonclassical histocompatibility antigen involved in immune tolerance, is expressed in breast carcinoma. Indeed, a majority of clinical breast cancers showed positive staining for HLA-G (Fig. 1a), and a quantitative analysis demonstrated that the HLA-G level was not associated with the expression of HER2, a specific biomarker for breast cancer subtyping (Fig. 1b). Analysis of 108 HER2-positive patients showed that HLA-G expression correlated with the degree of tumor differentiation and Tumor Node Metastasis staging, but no other characteristics of the breast cancer patients (Supplementary Table S1). Clinical breast cancers that express higher levels of HLA-G had a poorer prognosis and a higher probability of recurrence in patients (Fig. 1c and Supplementary Table S2). More importantly, another study involving 30 patients revealed that breast cancer expressing higher HLA-G exhibited lower response rates to trastuzumab treatment (Tables 1 and 2). HLA-G was also widely detected in breast cancer and gastric cancer cell lines independent of HER2 expression (Fig. 1d and Supplementary Fig. S1). We next probed whether HLA-G on HER2-positive breast cancer cells is involved in the escape from trastuzumab-mediated killing by immune cells. Human T cells and NK cells were isolated from peripheral blood mononuclear cells (PBMCs) of healthy donors and expanded in vitro (Supplementary Fig. S2a, b). We then cocultured these cells with a HER2-overexpressing breast cancer cell line, SK-BR-3 cells, and found that the characteristics of NK cells, including trastuzumab-induced IFN-γ secretion and rapid degranulation, as evidenced by CD107a externalization^24^, remained unchanged after expansion (Supplementary Fig. S2c, d). Trastuzumab induced the cytotoxicity of the NK cells, but not the T cells, in a coculture system with SK-BR-3 cells (Supplementary Fig. S3), which was in accordance with previous reports indicating that trastuzumab elicits the antitumor immunity of NK cells via ADCC.^25^ Knocking down HLA-G on SK-BR-3 cells improved the sensitivity to trastuzumab-mediated killing by NK cells without significantly affecting HER2 expression (Fig. 1e, f and Supplementary Fig. S4). Similarly, the HLA-G-blocking antibody remarkably enhanced the ADCC activity of NK cells with respect to HER2-positive cancer cells, but not those that negligibly expressed HER2 (Fig. 1g and Supplementary Fig. S1). The effects of HLA-G knockdown and blockade via an antibody on the cytotoxicity of cocultured NK cells were comparable, and knockdown of HLA-G failed to further enhance ADCC in cells previously blocked with antibody (Fig. 1h). These data suggest that HLA-G promotes HER2-positive breast cancer resistance to trastuzumab treatment by impairing ADCC.

**Table 1:** The association of HLA-G expression with trastuzumab response in patients with HER2-positive breast cancer (*n* = 30)

Total case number (*n* = 30)	HLA-G expression		*χ*^2^	*P* value
	Positive (*n* = 20)	Negative (*n* = 10)		
Trastuzumab-sensitive	6	9	9.6	0.001*
Trastuzumab-resistant	14	1		

*P < 0.05

**Table 2:** Clinical and pathological information for HER2-positive breast cancer patients treated with trastuzumab

Sample	Age	Biopsy	ER (IHC)	PgR (IHC)	HER2 (IHC)	AJCC stage	Surgery	Trastuzumab response
1	56	Ductal infiltrating carcinoma	+	+	+++	IIIC	Left radical mastectomy	Resistant
2	59	Ductal infiltrating carcinoma	++	++	+++	IIIA	Right radical mastectomy	Resistant
3	67	Ductal Infiltrating carcinoma	+	−	+++	IIIA	Right radical mastectomy	Resistant
4	52	Infiltrating lobular carcinoma	+	−	+++	IIIA	Left radical mastectomy	Resistant
5	54	Ductal infiltrating carcinoma	++	−	+++	IIIA	Right radical mastectomy	Resistant
6	66	Ductal infiltrating carcinoma	++	+	+++	IIIA	Right radical mastectomy	Resistant
7	56	Ductal infiltrating carcinoma	−	−	+++	IIIC	Left radical mastectomy	Resistant
8	49	Ductal infiltrating carcinoma	++	+	+++	IIA	Right radical mastectomy	Resistant
9	52	Ductal infiltrating carcinoma	−	−	+++	IIA	Right radical mastectomy	Resistant
10	53	Ductal infiltrating carcinoma	+	−	+++	IIIC	Right radical mastectomy	Resistant
11	47	Ductal infiltrating carcinoma	+	+	+++	IIIA	Left radical mastectomy	Resistant
12	62	Infiltrating carcinoma	−	−	+++	IIA	Right radical mastectomy	Resistant
13	55	Ductal infiltrating carcinoma	+	+	+++	IIA	Right radical mastectomy	Resistant
14	52	Ductal infiltrating carcinoma	−	−	+++	IIIC	Left radical mastectomy	Resistant
15	50	Ductal infiltrating carcinoma	++	+	+++	IIIA	Right radical mastectomy	Resistant
16	42	Ductal infiltrating carcinoma	+	+	+++	IIA	Right radical mastectomy	Sensitive
17	49	Ductal infiltrating carcinoma	++	−	+++	IIIC	Right radical mastectomy	Sensitive
18	45	Ductal Infiltrating carcinoma	+	−	+++	IIA	Right radical mastectomy	Sensitive
19	51	Ductal infiltrating carcinoma	−	−	+++	IIIA	Left radical mastectomy	Sensitive
20	40	Infiltrating lobular carcinoma	−	−	+++	IIIA	Left radical mastectomy	Sensitive
21	56	Ductal infiltrating carcinoma	+	+	+++	IIA	Left radical mastectomy	Sensitive
22	58	Infiltrating lobular carcinoma	++	−	+++	IIIC	Left radical mastectomy	Sensitive
23	55	Infiltrating carcinoma	−	−	+++	IIIA	Right radical mastectomy	Sensitive
24	59	Ductal infiltrating carcinoma	−	−	+++	IIIA	Right radical mastectomy	Sensitive
25	46	Ductal infiltrating carcinoma	+	−	+++	IIA	Right radical mastectomy	Sensitive
26	56	Infiltrating carcinoma	++	+	+++	IIIA	Left radical mastectomy	Sensitive
27	45	Ductal infiltrating carcinoma	++	+	+++	IIIA	Left radical mastectomy	Sensitive
28	58	Ductal infiltrating carcinoma	−	−	+++	IIA	Right radical mastectomy	Sensitive
29	52	Infiltrating carcinoma	+	+	+++	IIIA	Left radical mastectomy	Sensitive
30	50	Ductal infiltrating carcinoma	+	−	+++	IIA	Right radical mastectomy	Sensitive

### HLA-G impairs ADCC by binding to KIR2DL4 on NK cells

**Fig. 2:**
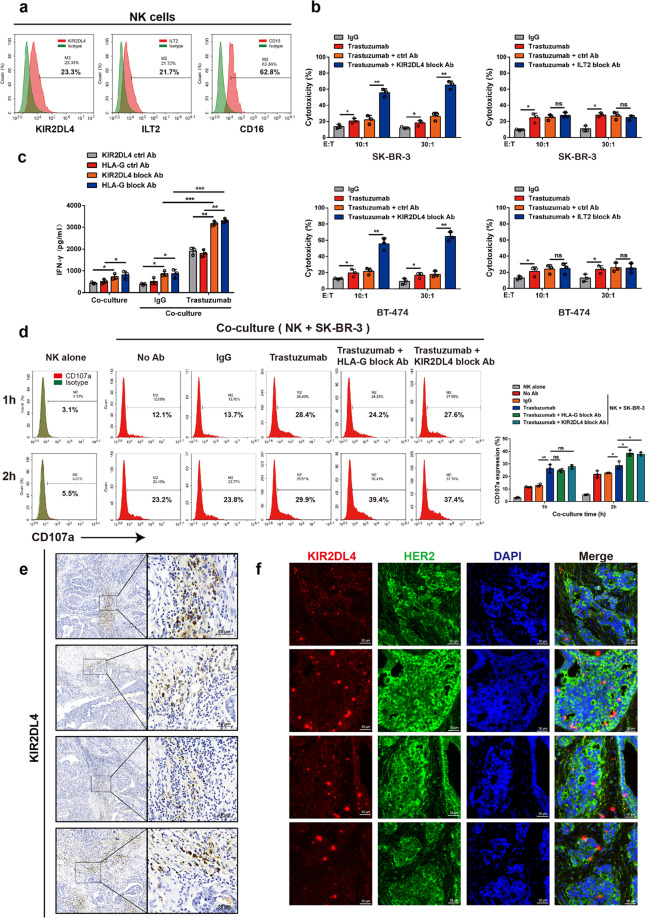
HLA-G engages KIR2DL4 on NK cells to suppress trastuzumab-elicited ADCC. **a** FCM assay of the expression of the indicated receptors on human PBMC-derived primary NK cells. **b** NK cells in the presence of trastuzumab were cocultured with HER2-overexpressing breast cancer cells at the indicated E:T ratios supplemented with or without the indicated blocking antibodies. The toxicity of NK cells to malignant cells was measured. **c**, **d** NK cells and SK-BR-3 cells were cocultured as described in (**b**) (E:T = 30:1). IFN-γ production was measured via ELISA (**c**), and the degranulation of NK cells was evaluated via FCM assay for CD107a expression (**d**). **e** Representative immunohistochemical staining of KIR2DL4 on infiltrating NK cells in clinical HER2-positive breast cancer tissues. **f** Representative immunofluorescent staining of HER2 and KIR2DL4 in breast cancer tissues of individual patients. All experiments were performed three times. Statistical significance was obtained by Student’s *t* test. *P < 0.05, **P < 0.01, and ***P < 0.001. n.s. Nonsignificant

HLA-G regulates innate immunity upon engagement of the NK cell receptor KIR2DL4 or the widely expressed leukocyte receptor Ig-like transcript 2 (ILT2)/CD85j.^26^ Consistently, we detected the expression of KIR2DL4, ILT2, and the type III Fcγ receptor (CD16) on expanded NK cells, indicating that this expression was not affected by the in vitro expansion protocol (Fig. 2a and Supplementary Fig. S5a, b). We then cocultured these cells with HER2-positive breast cancer cells and found that the blockade of KIR2DL4, but not that of ILT2, improved trastuzumab-mediated killing by NK cells to a level comparable to treatment with HLA-G antibody, suggesting that HLA-G exerts an inhibitory effect by binding to KIR2DL4 on NK cells (Fig. 2b and Supplementary Fig. S6a). We observed that the HLA-G- or KIR2DL4-neutralizing antibody, but not the ILT2 antibody, significantly increased IFN-γ production by NK cells cocultured with breast cancer cells in the presence of trastuzumab (Fig. 2c and Supplementary Fig. S6b). Blocking HLA-G or KIR2DL4 also promoted trastuzumab-induced degranulation of NK cells cocultured with HER2-positive breast cancer cells (Fig. 2d). The production of IFN-γ was not attributed to previously reported contamination of dendritic cells (DCs) during NK cell preparation or a response to IL-12 secreted by HLA-G-stimulated myeloid cells (Supplementary Fig. S7a, b).^27^ Histochemical assays verified widely distributed KIR2DL4 staining in HER2-positive breast cancer specimens, suggesting its abundant expression on infiltrating NK cells in tumor tissues (Fig. 2e, f). In addition, blocking HLA-G/KIR2DL4 enhanced the toxicity of NK cells to cocultured colorectal cancer cells induced by cetuximab, a monoclonal antibody against the epidermal growth factor receptor (EGFR), suggesting a common role of HLA-G/KIR2DL4 signaling in repressing ADCC in cancer immunotherapy (Supplementary Fig. S8a, b).^28^ Thus, HLA-G/KIR2DL4 mediates an interaction between malignant cells and NK cells resulting in repression of therapeutic antibody-triggered ADCC.

### Unengaged KIR2DL4 augments the cytotoxicity of NK cells by increasing IFN-γ production

**Fig. 3:**
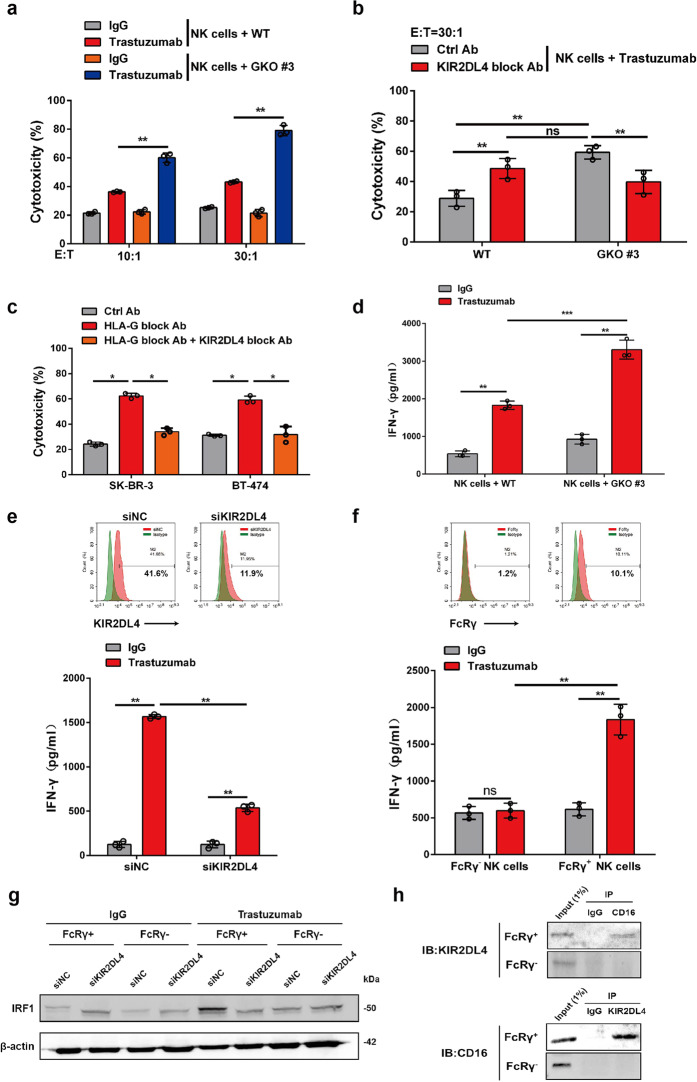
HLA-G-unbound KIR2DL4 promotes ADCC and IFN-γ production by NK cells. **a** NK cells in the presence of trastuzumab were cocultured with WT or HLA-G-knockout (GKO #3) SK-BR-3 cells supplemented with trastuzumab or IgG. The cytotoxicity of NK cells was measured via FCM. **b** NK cells were cocultured with WT or GKO #3 SK-BR-3 cells in the presence of trastuzumab alone or together with the indicated antibodies, and the cytotoxicity of NK cells was determined. **c** NK cells were cocultured with breast cancer cells in the presence of trastuzumab and the indicated blocking antibodies, and the cytotoxicity of NK cells was measured by FCM (E:T = 30:1). **d** NK cells were cocultured with WT or GKO #3 SK-BR-3 cells (E:T = 1:1) supplemented with trastuzumab or IgG. IFN-γ production was measured via ELISA. **e** NK cells transfected with control or KIR2DL4 siRNAs were subjected to FCM assay (upper). Cells were cocultured with GKO #3 SK-BR-3 cells and treated with IgG or trastuzumab, and IFN-γ production was measured via ELISA (lower). **f**–**h** NK cells from healthy donors were subjected to FACS according to FcRγ expression (**f**, upper). The FcRγ^+^ and FcRγ^-^ cells were then cocultured with GKO #3 SK-BR-3 cells plus treatment with IgG or trastuzumab, and were subjected to enzyme-linked immunosorbent assay (ELISA) (**f**, lower), Western blot analysis (**g**), and immunoprecipitation assay (**h**). All experiments were performed three times. Statistical significance was determined by Student’s *t* test. *P < 0.05, **P < 0.01, and ***P < 0.001. n.s. Nonsignificant

The regulatory role of KIR2DL4 in NK cell function remains controversial since it has both an ITIM in the cytoplasmic tail and an arginine–tyrosine motif in the transmembrane region required for the activation signal.^12^ We next probed how KIR2DL4 affects trastuzumab-induced ADCC in the absence of HLA-G. *HLA-G*-knockout SK-BR-3 cells were generated via CRISPR/Cas9-mediated gene depletion (Supplementary Fig. S9a–d). While these cells exhibited similar growth characteristics and HER2 expression as the parental cells (Supplementary Fig. S9e, f), they were more susceptible to killing by NK cells when incubated with trastuzumab (Fig. 3a). Further blockade of KIR2DL4 on NK cells attenuated their cytotoxicity against *HLA-G*-depleted SK-BR-3 cells (Fig. 3b). These results were in agreement with the observation using parental SK-BR-3 cells showing that concomitant neutralization of HLA-G and KIR2DL4 was less effective in sensitizing parental SK-BR-3 cells to trastuzumab-elicited killing than blocking HLA-G alone (Fig. 3c). While the ablation of HLA-G in SK-BR-3 cells significantly improved trastuzumab-induced IFN-γ production by cocultured NK cells (Fig. 3d), this effect was attenuated by further knockdown of KIR2DL4 in the NK cells (Fig. 3e). Next, we investigated how KIR2DL4 collaborates with trastuzumab to promote IFN-γ expression in NK cells. FcRγ, a signal-transducing adaptor for Fc receptors, was reported to mediate activating signals from IgG cross-linked with CD16/FcγRIII on the surface of NK cells.^9^ We thus proposed that FcRγ might license the interplay between KIR2DL4 and antibody-bound CD16. Indeed, the expression of FcRγ on tumor-infiltrating NK cells was validated using clinical breast cancer specimens (Supplementary Fig. S10). We then sorted NK cells prepared from healthy donors according to FcRγ expression, and found that trastuzumab induced the production of more IFN-γ by FcRγ-positive NK cells than by those expressing negligible levels of FcRγ (Fig. 3f). Similarly, IFN-γ production by FcRγ-expressing NK cells was also induced by cetuximab when cocultured with EGFR-positive malignant cells, suggesting a common mechanism of NK cell activation by antibody engagement of the Fcγ receptor CD16 (Supplementary Fig. S11). KIR2DL4 maintained high expression of interferon regulatory factor-1 (IRF1), a key factor transcriptionally activating *IFNG*, in a trastuzumab- and FcRγ-dependent manner (Fig. 3g).^29^ The association of KIR2DL4 with CD16 was verified in trastuzumab-incubated NK cells that express high levels of FcRγ (Fig. 3h). These data suggest that KIR2DL4, due to the lack of an ITAM, transduces activating signals through an FcRγ-mediated interaction with CD16.

### HLA-G and KIR2DL4 are upregulated by autocrine cytokines

**Fig. 4:**
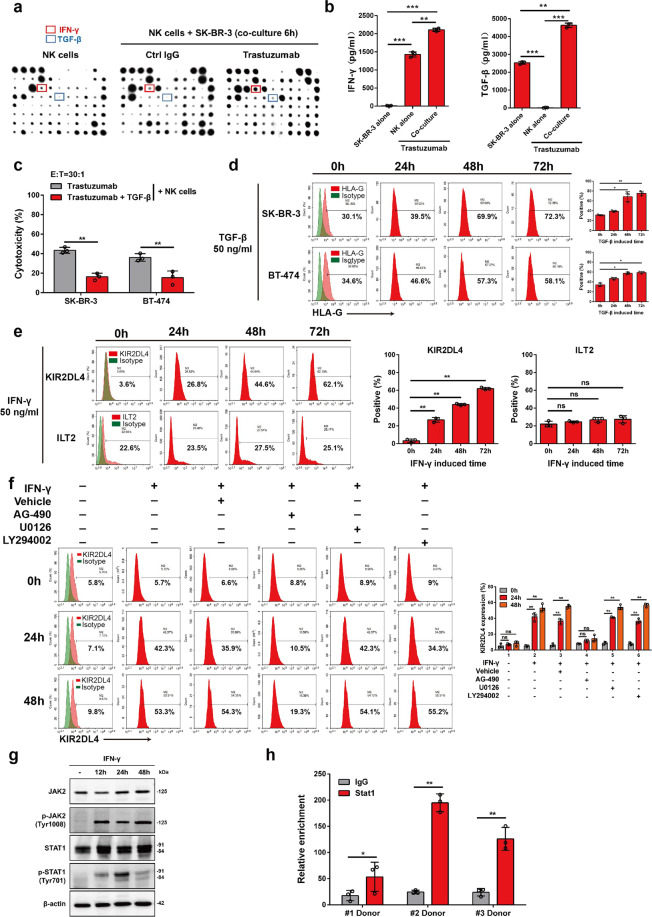
TGF-β and IFN-γ upregulate HLA-G on breast cancer cells and KIR2DL4 on NK cells, respectively. **a** NK cells were cultured alone or with SK-BR-3 cells (E:T = 1:1) supplemented with the indicated antibodies. The supernatant was harvested and measured for cytokine levels using a cytokine antibody array. **b** NK cells and SK-BR-3 cells were cocultured or cultured alone for 48 h with or without trastuzumab, followed by measurement of IFN-γ and TGF-β production via ELISA. **c** FCM assay for determining the trastuzumab-elicited cytotoxicity of NK cells to cocultured HER2-positive breast cancer cells when treated with or without TGF-β (50 ng/ml) for 6 h. **d** Cells were incubated with TGF-β for the indicated times and were subjected to FCM assay for determining HLA-G level. **e**, **f** FCM assay for the expression of KIR2DL4 and ILT2 on NK cells incubated with IFN-γ and different inhibitors for the indicated times. **g** NK cells were incubated with IFN-γ (50 ng/ml) for the indicated times and were subjected to Western blot analysis. **h** ChIP assay for determining the enrichment of STAT1 on the KIR2DL4 promoter using NK cells prepared from different donors. All experiments were performed three times. Statistical significance was obtained by Student’s *t* test. *P < 0.05, **P < 0.01, and ***P < 0.001. n.s. Nonsignificant

We next examined whether the trastuzumab-mediated interplay between HER2-overexpressing breast cancer cells and NK cells regulates HLA-G and KIR2DL4 expression. Cytokines play important roles in mediating the cross-talk between different types of cells in the tumor microenvironment.^30^ Cytokine profiles produced by cocultured cells were then tested, which showed that trastuzumab increased the production of IFN-γ and tumor growth factor-β (TGF-β) (Fig. 4a). We next performed ELISA to determine the source of these cytokines. We found that IFN-γ was mainly secreted by NK cells, and TGF-β was produced predominantly by tumor cells (Fig. 4b). The production of both cytokines was enhanced when the cells were cocultured in the presence of trastuzumab (Fig. 4b). TGF-β significantly impaired the trastuzumab-mediated cytolytic activity of NK cells cocultured with HER2-positive breast cancer cells (Fig. 4c). In line with this finding, we observed that HLA-G was upregulated on tumor cells treated with TGF-β, but not those with IFN-γ (Fig. 4d and Supplementary Fig. S12a). In addition, IFN-γ but not TGF-β treatment of NK cells induced a significant increase in the level of KIR2DL4, but not the level of ILT2 (Fig. 4e and Supplementary Fig. S12b). IFN-γ can activate various intracellular signaling pathways, including the JAK/STAT, PI3K/Akt, and ERK pathways.^31–33^ However, we found that AG490, a specific JAK2 inhibitor, but not the PI3K inhibitor Ly294002 or the MAPK/ERK inhibitor U0126, abrogated IFN-γ-induced upregulation of KIR2DL4 on NK cells (Fig. 4f). Consistently, JAK2/STAT1 signaling was remarkably activated in IFN-γ-stimulated NK cells (Fig. 4g). A chromatin immunoprecipitation (ChIP) assay confirmed the enrichment of STAT1 on the *KIR2DL4* promoter (Fig. 4h), suggesting that IFN-γ transcriptionally activates KIR2DL4 via JAK2/STAT1 signaling. These data indicate that trastuzumab may upregulate HLA-G on malignant cells and KIR2DL4 on NK cells by orchestrating cytokine production in HER2-positive breast cancer.

### Paracrine cytokines mediate cross-talk between KIR2DL4 and PD-L1/PD-1 signaling pathways

**Fig. 5:**
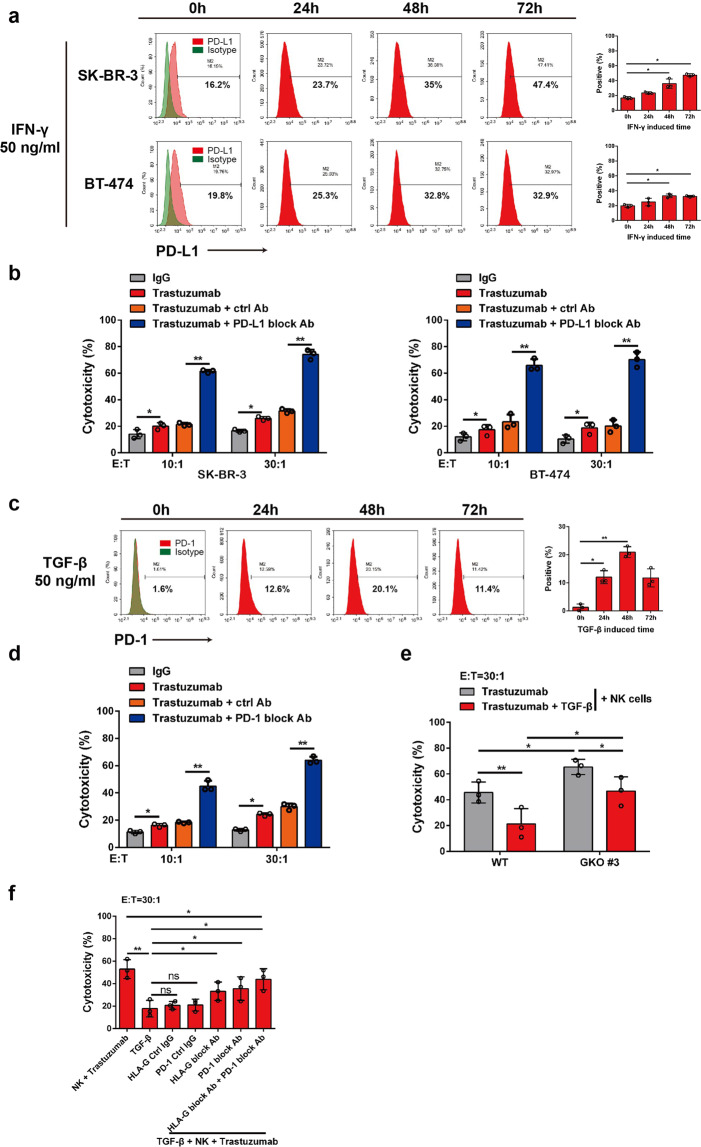
TGF-β and IFN-γ repress trastuzumab-elicited ADCC by upregulating PD-L1/PD-1. **a** FCM assay for determining the expression of PD-L1 on breast cancer cells incubated with IFN-γ for the indicated times. **b** NK cells were cocultured with breast cancer cells supplemented with control or PD-L1-blocking antibody, and the cytotoxicity of NK cells was measured by FCM. **c** FCM assay for determining the expression of PD-1 on NK cells incubated with TGF-β for the indicated times. **d** NK cells cocultured with SK-BR-3 cells were treated with TGF-β (50 ng/ml) for 6 h. Trastuzumab together with a control or PD-1-blocking antibody was then added, and the cytotoxicity of the NK cells was measured by FCM. **e** NK cells were cocultured with WT or GKO #3 SK-BR-3 cells in the presence of trastuzumab, and were treated with or without TGF-β (50 ng/ml) for 6 h, and a cytotoxicity assay was performed via FCM. **f** NK cells cocultured with wild-type SK-BR-3 cells in the presence of trastuzumab were treated with or without TGF-β (50 ng/ml, 6 h) and/or incubated with the indicated antibodies, and then a cytotoxicity assay was performed via FCM. All experiments were performed three times. Statistical significance was obtained by Student’s *t* test. *P < 0.05 and **P < 0.01. n.s. Nonsignificant

The multifaceted functions of cytokines prompted us to explore whether TGF-β and IFN-γ also act in a paracrine manner in the context of breast cancer cell and NK cell interactions. A recent study showed that the documented immune checkpoint PD-L1/PD-1 is critically involved in trastuzumab resistance in breast cancer.^34,35^ Consistent with previous reports, we observed that IFN-γ significantly increased the level of PD-L1 in breast cancer cells (Fig. 5a).^36^ Blockade of PD-L1 increased the cytotoxicity of NK cells against trastuzumab-treated HER2-overexpressing breast cancer cells (Fig. 5b). In parallel, TGF-β treatment significantly upregulated PD-1 on NK cells, and blocking PD-1 enhanced trastuzumab-mediated ADCC of NK cells against human breast cancer SK-BR-3 cells (Fig. 5c, d). These results were in accordance with the observation that TGF-β treatment compromised the trastuzumab-induced killing of breast cancer cells even when the *HLA-G* gene was ablated (Fig. 5e). However, TGF-β might also impair trastuzumab-induced ADCC via other mechanism(s) since combined neutralization of HLA-G and PD-L1 failed to completely rescue the cytotoxicity of NK cells suppressed by TGF-β (Fig. 5f). These results, together with previous findings that KIR2DL4 forms a regulatory circuit with IFN-γ, suggest complicated cross-talk between different immune checkpoints mediated by cytokines in trastuzumab-treated breast cancer.

### Blockade of HLA-G signaling enhances the antitumor activity of trastuzumab in vivo

**Fig. 6:**
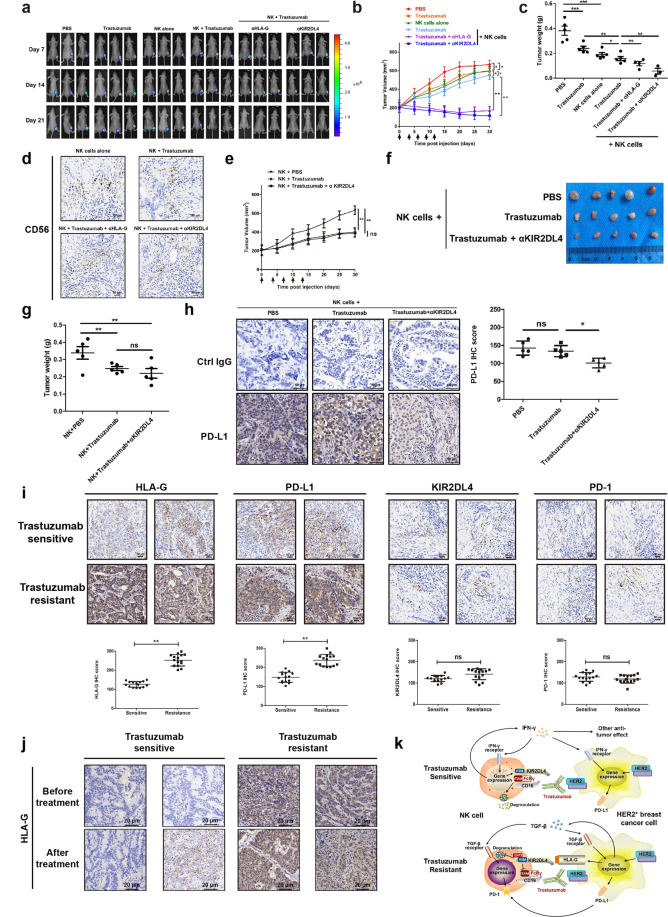
HLA-G/KIR2DL4 signaling impairs the in vivo antitumor activity of NK cells mediated by trastuzumab. **a** Nude mice were inoculated with luciferase-expressing SK-BR-3 cells. When the tumor volume reached 200 mm^3^ (day 0), mice were subjected to intraperitoneal treatment with 1 × 10^7^ primary NK cells, trastuzumab (5 mg/kg), and KIR2DL4/HLA-G-blocking antibodies (1 mg/kg) on days 0, 3, 6, 9, and 12. Bioluminescence imaging was performed on the indicated days. **b** Volumes of the tumors in the mice as described in (**a**) were monitored and plotted. Arrows indicated days when treatment was conducted. **c** Mice were sacrificed on day 30, and tumors were excised and weighed. **d** Tumors in (**b**) were sectioned and subjected to immunohistochemical staining. **e** Nude mice were inoculated with GKO #3 SK-BR-3 cells. When the tumor volume reached 200 mm^3^ (day 0), mice were subjected to intraperitoneal treatment with 1 × 10^7^ primary NK cells, trastuzumab (5 mg/kg), and KIR2DL4-blocking antibody (1 mg/kg) on days 0, 3, 6, 9, and 12. Volumes of the tumors were monitored and plotted. **f**, **g** Mice in (**e**) were sacrificed on day 30, and tumors were excised and weighed. **h** Representative immunohistochemical staining and IHC scores of PD-L1 in xenograft tumor tissues as shown in (**f**). **i** Representative immunohistochemical staining and IHC scores of HLA-G, PD-L1, KIR2DL4, and PD-1 in post-treatment breast cancer tissues collected from trastuzumab-sensitive and trastuzumab-resistant individual patients. **j** Immunohistochemical staining of HLA-G was performed on paired pre- and post-treatment specimens collected from HER2-positive breast cancer patients. **k** Schematic diagram for the regulatory roles of HLA-G and KIR2DL4 in trastuzumab-induced ADCC. In the absence of HLA-G, KIR2DL4 forms a feedback circuit with IFN-γ to activate NK cells, which is potentially compromised by IFN-γ upregulation of PD-L1 on neoplastic cells. HLA-G engagement of KIR2DL4 inactivates NK cells. Although not always occurring simultaneously, PD-1 upregulation by tumor-derived TGF-β might coordinate with HLA-G to repress the cytotoxicity of NK cells. All experiments were performed three times. Statistical significance was obtained by Student’s *t* test. *P < 0.05, **P < 0.01, and ***P < 0.001. n.s. Nonsignificant

To assess the in vivo antitumor activity of NK cells, a xenograft tumor model was established by inoculation of nude mice with SK-BR-3 cells modulated to express firefly luciferase. Bioluminescent imaging showed that tumors significantly shrank upon intraperitoneal injection of human NK cells and trastuzumab, compared with injection of NK cells or trastuzumab alone (Fig. 6a–c). Blockade of HLA-G signaling using specific antibodies against HLA-G and KIR2DL4 further enhanced the capability of NK cells and trastuzumab to repress HER2-positive tumors (Fig. 6a–c). Immunohistochemical (IHC) analysis revealed apparent infiltration of CD56^+^ NK cells into the tumor tissue (Fig. 6d). Antibodies against HLA-G/KIR2DL4 combined with trastuzumab and NK cells, but not blocking antibodies alone, suppressed tumor growth in vivo, suggesting that these blocking antibodies exert a tumoricidal role at least partially by enhancing ADCC activity (Supplementary Fig. S13). We next generated xenograft breast cancer models using *HLA-G*-null SK-BR-3 cells. In contrast to the observation of tumors derived from parental SK-BR-3 cells, blocking KIR2DL4 failed to remarkably alter the suppressive efficacy of NK cells and trastuzumab in these tumors (Fig. 6e–g). Nonetheless, KIR2DL4 neutralization reduced PD-L1 levels in tumor tissues (Fig. 6h), a finding in agreement with the aforementioned report that KIR2DL4 unassociated with HLA-G promoted IFN-γ secretion by NK cells and that IFN-γ upregulated PD-L1 on tumor cells (Figs. 3e and 5a). Finally, we probed the role of HLA-G/KIR2DL4 in the response of clinical breast cancer to trastuzumab using neoplastic specimens of trastuzumab-resistant and trastuzumab-sensitive patients (Table 2). We detected higher levels of HLA-G and PD-L1 in the trastuzumab-resistant tumors than those sensitive to trastuzumab treatment (Fig. 6i). We also detected the coordinated expression of KIR2DL4 and PD-1 in these tumor tissues (Fig. 6i). The development of trastuzumab resistance was coordinated by a remarkable upregulation of HLA-G as observed in clinical HER2-positive breast cancer samples (Fig. 6j). These results showed that the HLA-G/KIR2DL4 interaction can promote breast cancer resistance to trastuzumab in vivo by circumventing NK cell killing and that KIR2DL4 on tumor-infiltrating NK cells can play different roles depending on the availability of HLA-G.

## Discussion

The frequent development of drug resistance poses a major barrier to the widespread use of trastuzumab in clinical breast cancer treatment.^37^ Consistent with the essential role of ADCC in the therapeutic efficacy of trastuzumab, the development of trastuzumab resistance correlates with compromised NK cell cytotoxicity.^38^ Nonetheless, it remains unclear how ADCC is regulated in trastuzumab-treated breast cancer. Here, we established that the abundant expression of HLA-G on breast cancer cells and its engagement with the atypical KIR family receptor, KIR2DL4, on NK cells suppress ADCC and contribute to breast cancer resistance to trastuzumab. While KIR2DL4 alone synergizes with FcRγ to enhance NK cell activation and degranulation, HLA-G binding to KIR2DL4 impairs the cytotoxicity of NK cells. In agreement with these observations, we found that blocking the HLA-G/KIR2DL4 interaction resensitized breast cancer to trastuzumab treatment (Fig. 6k).

While the role of KIR2DL4 in the NK cells in the tumor microenvironment remains uncharacterized, we found that, in the absence of HLA-G, KIR2DL4 forms a feedback circuit with IFN-γ to promote trastuzumab-induced ADCC in vitro. These observations are similar to those of CD80/CD86 on T cells, which are activated and inhibited by CD28 and CTLA4, respectively, and function as a classical immune checkpoint, suggesting that KIR2DL4 might provide a switch for NK cell activity via its association with HLA-G.^39^ Further studies using the protein interactome and loss-of-function technologies are needed to determine whether the activation of NK cells via KIR2DL4 is ligand-independent. In addition, we failed to observe a net decrease in the immunological response against HLA-G-deficient tumors in vivo upon blockade of KIR2DL4, probably because KIR2DL4 and IFN-γ mediate extensive interplay between neoplastic and stromal cells in the tumor microenvironment, although the specific regulatory mechanisms remain unknown. Therefore, our study suggests novel approaches to improving trastuzumab efficacy in HER2-positive breast cancer patients, e.g., antibody- or CRISPR/Cas9-mediated depletion of HLA-G, or development of soluble KIR2DL4 or blockers for the HLA-G/KIR2DL4 interaction.

An immunosuppressive microenvironment exemplified by a rewired cytokine network and upregulated immune checkpoint proteins is a hallmark of advanced and therapy-refractory tumors.^40^ Here, we found that trastuzumab increased the production of TGF-β and IFN-γ by breast cancer cells and NK cells, respectively. Consistent with previous reports on the regulation of PD-1 signaling and the established role of TGF-β in promoting cancer resistance to trastuzumab^41,42^, we observed that PD-1 on NK cells was induced by TGF-β, and PD-1 blockade significantly increased the cytotoxicity of NK cells. In addition, autocrine TGF-β signaling in tumor cells also promoted the expression of HLA-G, which bound to KIR2DL4 and enabled tumor cells to inhibit the activation of NK cells. Although IFN-γ has been reported to upregulate the immune checkpoint ligand PD-L1 in cancer cells, it is also involved in boosting antitumor immunity induced by therapeutic antibodies.^43,44^ Consistent with these results, we demonstrated a dual role of IFN-γ derived from activated NK cells in trastuzumab-induced antitumor immunity. IFN-γ forms a regulatory circuit with KIR2DL4 to improve the cytotoxicity of NK cells. However, IFN-γ also induces PD-L1 expression in human HER2-overexpressing breast cancer cells. Together, these findings suggest the necessity of combined HLA-G and PD-L1/PD-1 targeting for the effective treatment of trastuzumab-resistant breast cancer.

## Materials and methods

### Cell culture and reagents

Human breast cancer cell lines (SK-BR-3, BT-474, MDA-MB-231, and MCF-7 cells), human gastric cancer cell lines (SGC-7901 and NCI-N87 cells), human colorectal cancer cell lines (HT29, SW480, and LoVo cells), and human embryonic kidney 293T (HEK293T) cells were obtained from the American Type Culture Collection (Manassas, USA). Luciferase-expressing recombinant lentiviruses (GeneChem, Shanghai, China) were used to achieve stable luciferase expression in SK-BR-3 cells. HEK-293T and SK-BR-3-luciferase cells were maintained in Dulbecco’s modified Eagle’s medium (Thermo Fisher, Waltham, USA) supplemented with 10% heat-inactivated fetal bovine serum (Thermo Fisher). All human breast cancer and gastric cancer cell lines were cultured in RPMI-1640 (Thermo Fisher). Human colorectal cancer HT29 cells were cultured in McCoy’s 5a medium, SW480 cells were cultured in L-15 medium, and LoVo cells were cultured in F-12K medium. All cells were supplemented with 100 U/ml penicillin and 100 μg/ml streptomycin (Invitrogen, Carlsbad, USA) at 37 °C in a humidified 5% CO_2_ incubator.

### Patients

For examination of HLA-G expression in clinical specimens, tumor arrays were obtained from Shanghai Outdo Biotech Company (Cat. No. HBreD140Su04, Shanghai, China). All patients received surgery without chemotherapy or radiotherapy before surgery, and the histological types and clinical stages of tumors were classified based on the criteria of the American Journal of Critical Care. For the analysis of the correlation of HLA-G expression with patient survival, eligible patients were at least 18 years old with a histologically confirmed diagnosis of HER2 positivity (IHC score ≥3, or IHC score ≥2 and FISH+). The inclusion criteria were adequate bone marrow, hepatic, and renal function; a minimum survival of 3 months; and signed consent. Exclusion criteria included having incomplete case information, dying of complications after surgery, and coexistence of other malignancies. Tumor tissue samples were collected from the Department of Oncology of the Second Affiliated Hospital of Xi’an Jiaotong University from January 2017 to June 2020 for the analysis of the correlation of HLA-G expression with patient response to trastuzumab treatment. The inclusion and exclusion criteria were the same as those for correlation analysis of HLA-G expression with patient survival, except that the patients received routine neoadjuvant trastuzumab and chemotherapy (TCbH or EC-TH regimen) for 18–24 weeks, but not radiotherapy or other therapeutics that might affect the immune system. All specimens were obtained as described elsewhere.^45^ Patients were then subjected to biopsy once they reached the criteria of partial response (PR), which were defined as at least a 30% decrease in the sum of the longest tumor diameter compared to the baseline that was maintained for a minimum of 4 weeks. Then, modified radical mastectomy was performed, followed by trastuzumab maintenance therapy (for as many as 52 weeks). Patients with a complete response, i.e., the disappearance of all target lesions in a computed tomography (CT) scan, were considered to be trastuzumab-sensitive, and the specimens obtained when PR was first confirmed were used as post-treatment samples for these patients. In contrast, those with progressive disease, as verified by CT scanning, during continued trastuzumab treatment were considered to be trastuzumab-resistant. For these patients, biopsies were performed at the time of progression, and the specimens were used as post-treatment samples.

### Isolation and analysis of tumor-infiltrating NK cells

Fresh tumors were obtained from HER2-positive breast cancer patients undergoing surgery, and were minced and mechanically dissociated with the GentleMACS Tumor Dissociation Kit (Miltenyi Biotec, Bergisch Gladbach, Germany) according to the manufacturer’s instructions. Cells were subjected to flow cytometry (FCM) for NK cells using phycoerythrin (PE)-labeled CD56 (Cat. No. 362507, BioLegend), peridinin–chlorophyll–protein-conjugated CD3 (Cat. No. 300325, BioLegend), and fluorescein isothiocyanate (FITC)-labeled FcRγ (Cat. No. FCABS400F, Sigma-Aldrich) antibodies. The CD56^+^CD3^−^ cells were then gated and examined for FcRγ expression.

### In vitro NK cell and T cell expansion

NK cells and T cells were isolated from the peripheral blood of healthy adult donors. Written informed consent was obtained from all participants. PBMCs were isolated by Ficoll density gradient centrifugation, and NK cells were purified by negative magnetic selection using an NK Cell Isolation Kit (Miltenyi) following the manufacturer’s instructions. T cells were negatively purified by the MojoSort™ Human CD3 T Cell Isolation Kit (BioLegend) following the manufacturer’s instructions. The purity of the isolated cell subsets was assessed by FCM using the aforementioned CD56 and CD3 antibodies, and cells were also examined for possible contamination of DCs via FCM. T cells were expanded for 3 days in SuperCulture™ L500 (Cat. No. 6111021, Dakewei) supplemented with 1 μg/ml LEAF™ purified anti-human CD3/CD28 (Cat. Nos. 317326 and 302934, BioLegend) and 15 ng/ml IL-2 (Cat. No. 200-02, PeproTech). Then, T cells were maintained in the same medium, and 15 ng/ml IL-2 was added every other day. NK cells were expanded in α-MEM medium (Cat. No.12571-063, Gibco) supplemented with 15% fetal bovine serum and 10 ng/ml anti-CD3 antibody (Cat. No. GTX79905, GeneTex), and 50 ng/ml IL-15 (Cat. No.200-15, PeproTech) and 15 ng/ml IL-2 (Cat. No.200-02, PeproTech) were added every other day. Cells from expansion days 14–21 with purity >90% were used for further experiments. The expanded NK cells were also subjected to fluorescence-activated cell sorting (FACS) according to FcRγ expression using a FITC-conjugated antibody (Cat. No. FCABS400F, Sigma-Aldrich).

### Cytokine antibody array

Human breast cancer cell lines were plated in 6-well plates and cocultured with human primary NK cells at a 1:1 ratio. After 6 h, the cell-free culture supernatant was collected for examination of cytokine production using the Human Cytokine Antibody Array C5 (RayBiotech, Peachtree Corners, USA). Briefly, cytokine antibody array membranes were blocked with blocking buffer for 30 min. The membranes were incubated with cell-free culture supernatant containing equal amounts of proteins, followed by incubation with biotin-labeled antibodies against various individual cytokines. The membranes were then washed and incubated with horseradish peroxidase (HRP)-conjugated streptavidin for 2 h. Unbound HRP–streptavidin was washed off with wash buffer I and wash buffer II. Finally, the signals were detected and densitometry values of the cytokines were quantified.

### ELISA

Human IFN-γ and TGF-β levels in the cell culture supernatant were measured using commercially available ELISA Kits (Dakewei, Shenzhen, China) following the manufacturer’s instructions. Briefly, the conditioned medium from the cell cultures was added to an ELISA Kit plate that was precoated with a specific antibody. A biotinylated secondary antibody was then added and the plate was incubated at room temperature for 2 h. The color development catalyzed by HRP was terminated with 2.5 mol sulfuric acid, and the absorption was measured at 450 nm. The protein concentration was normalized to the relative absorbance rate of the respective standard and expressed as the mean ± SD.

### Flow cytometry

Primary NK cells and breast cancer cell lines were analyzed via FCM using antibodies as follows: PE-labeled anti-human CD3 (Cat. No. 561808, BD Biosciences, Heidelberg, Germany), FITC-labeled anti-human CD8 (Cat. No. 557085, BD Biosciences), FITC-labeled anti-human CD56 (Cat. No. 562794, BD Biosciences), PE-labeled anti-human CD16 (Cat. No. 556619, BD Biosciences), PE-labeled anti-human KIR2DL4 (Cat. No. MA1-10111, eBioscience, San Diego, USA), PE-labeled anti-human ILT2 (Cat. No. 12-5129-42, eBioscience), PE-labeled anti-human HLA-G (Cat. No. MA1-10369, eBioscience), FITC-labeled anti-human PD-1 (Cat. No. 11-9969-42, eBioscience), PE-labeled anti-human PD-L1 (Cat. No. 12-5983-41, eBioscience), FITC-labeled anti-human CD107a (Cat. No. 11-1079-42, eBioscience), FITC-conjugated FcRγ antibody (Cat. No. FCABS400F, Sigma-Aldrich), and PE-conjugated CD11c antibody (Cat. No. 371503, BioLegend). Unspecific antibody binding was analyzed by staining with isotype-matched FITC- and PE-labeled control antibodies (BD Biosciences). Briefly, 5 × 10^5^ cells were washed and resuspended in phosphate-buffered saline (PBS) buffer containing 2% bovine serum albumin and 0.2% sodium azide and incubated with either directly labeled antibody or unconjugated antibody (followed by directly labeled secondary antibody) for 30 min at 4 °C. After incubation, the cells were washed three times and analyzed using FCM (BD Bioscience).

### siRNA synthesis and transfection

The small interfering RNAs (siRNAs) targeting HLA-G and KIR2DL4 were synthesized by GenePharma (Shanghai, China). The siRNAs were transfected into cells using Lipofectamine 2000 (Invitrogen) according to the manufacturer’s instructions. The following sequences of siRNA duplex sense strands were used: siHLA-G #1, 5′-GCAGAGAUACACGUGCCAUTT-3′; siHLA-G #2, 5′-CCGCGGGUAUGAACAGUAUTT-3′; siKIR2DL4 #1, 5′-GATCATGGTCACAGGTCTA-3′; siKIR2DL4 #3, 5′-TCACAGGTCTATATGAGAA-3′; and negative control, 5′-UUCUCCGAACGUGUCACGUTT-3′. For the in vitro study, cells were harvested for analysis 48 h after transfection with duplex siRNA.

### Generation of HLA-G-knockout cells

HLA-G-knockout SK-BR-3 cells were generated via CRISPR/Cas9 technology. Single guide RNAs (sgRNAs) were designed using an online tool (http://crispr.mit.edu). The sgRNA coding oligos (#1: 5′-CACCGACAGCGACTCGGCGTGTCCG-3′; #2: 5′-CACCGGGTCGCAGCCAATCATCCAC-3′; and #3: 5′-CACCGTCATTCTGTCAGTCTGTGCG-3′) were cloned into an sgRNA expression vector lentiCRISPR v2 (Cat. No. 52961, Addgene, Cambridge, USA). SK-BR-3 cells were transfected with an sgRNA-HLA-G expression vector using Lipofectamine 2000 (Invitrogen) and selected using puromycin (0.5 µg/ml). HLA-G knockout was validated by Western blotting, reverse transcription-polymerase chain reaction, and FCM. After 2 weeks of screening, the mixed clones were transferred into 96-well plates using limited dilution to obtain monoclonal cells (GKO #1, #2, and #3). HLA-G^+^ SK-BR-3 cells from the same culture were used as controls.

### MTT assay

Cell growth was measured using 3-(4, 5-dimethylthiazol-2-yl)-2, 5-diphenyltetrazolium bromide (MTT) reagent (Sigma-Aldrich, St. Louis, USA). Briefly, 4 × 10^3^ cells in each group were plated in 96-well plates with 200 μl of medium per well. Twenty microliters of the MTT in PBS (2.5 mg/ml) was added to each well. The plates were then placed back in the incubator. Four hours later, the medium was removed, and the cells were solubilized in 150 μl of dimethylsulfoxide before colorimetric analysis (wavelength, 490 nm). One plate was analyzed after adhesion of the cells (~4 h after plating), and the other plates were measured over the next 4 consecutive days.

### Quantitative reverse transcription PCR

Total RNA was extracted using TRIzol reagent (Invitrogen) following the manufacturer’s instruction. RNA was reverse transcribed using the Prime-Script RT Reagent Kit (TaKaRa, Kusatsu, Japan). Quantitative PCR was conducted using a CFX96TM Real-Time PCR system (Bio-Rad, Hercules, USA) with SYBR Green Reagents (TaKaRa). Gene expression levels were normalized to that of glyceraldehyde 3-phosphate dehydrogenase (GAPDH). The primers used for real-time PCR were as follows: HLA-G-F, 5′-CGTCCTGGGTCTGGTCCT-3′; HLA-G-R, 5′-GTGGCTCCACAGATACCTG-3′; GAPDH-F, 5′-GGTGAAGGTCGGTGTGAACG-3′; and GAPDH-R, 5′-CTCGCTCCTGGAAGATGGTG-3′.

### Chromatin immunoprecipitation

Cells were cross-linked with formaldehyde and harvested for ChIP. Briefly, chromatin was fragmented by sonication, and precleared chromatin was immunoprecipitated overnight with STAT1 antibody (Cat. No. 9172, Cell Signaling Technology, Boston, USA) or IgG as a negative control. The enrichment of specific DNA fragments was analyzed by PCR. The primers used for amplification of the human *KIR2DL4* promoter region were as follows: 5′-AATACATCAAATTTCCTCATGTGA-3′ and 5′-TCTGCTGCCAGGACGCAGTGA -3′.

### Immunoprecipitation assay

NK cells were collected using Western blot and IP buffers (Beyotime, Jiangsu, China) containing PMSF protease inhibitor according to the manufacturer’s instructions. Whole-cell extract (30 μl) was used as input, and the remainder was divided into two equal parts and incubated separately with an IgG control and an anti-CD16 (Cat. No. ab246222, Abcam) or anti-KIR2DL4 (Cat. No. GTX80038, GeneTex) antibody at 4 °C overnight with gentle rotation. Protein G magnetic beads (Invitrogen) were added to the lysates and incubated for 2–3 h according to the manufacturer’s instructions. Precipitates were washed five times with TBST buffer, and the proteins were eluted by boiling the beads for 5 min in 1× sodium dodecyl sulfate (SDS) sample buffer. Eluted proteins and input whole-cell extracts were analyzed by Western blotting.

### Western blot analysis

Cells were lysed in lysis buffer (50 mM NaCl, 50 mM EDTA, and 1% Triton X-100) supplemented with a protease inhibitor cocktail (Roche, Basel, Switzerland). Cell lysates were separated by SDS-polyacrylamide gel electrophoresis and transferred to nitrocellulose membranes. Membranes were then blocked with 5% nonfat milk diluted in PBS for 2 h at room temperature and incubated with the following primary antibodies: anti-HLA-G (Cat. No. ab169778, Abcam, Cambridge, UK); anti-JAK2 (Cat. No. 3230, Cell Signaling Technology); anti-STAT1 (Cat. No. 9172, Cell Signaling Technology); anti-phosphorylated JAK2 (Cat. No. 8082, Cell Signaling Technology); anti-phosphorylated-STAT1 (Cat. No. 9167, Cell Signaling Technology); anti-IRF-1 (Cat. No. 8478, Cell Signaling Technology); and anti-β-actin (Cat. No. A4700, Sigma-Aldrich). Then, membranes were washed with PBS containing 0.05% Tween and incubated with secondary antibodies conjugated with HRP for 1 h at room temperature. The bands were developed using a chemiluminescence reagent (Thermo Fisher).

### ADCC assay

NK cell cytotoxicity was measured via FCM as described previously.^46^ Briefly, isolated NK cells were incubated with carboxyfluorescein succinimidyl ester (CFSE)-labeled SK-BR-3 cells at different effector/target ratios at 37 °C and 5% CO_2_. Trastuzumab (0.105 mg/ml), cetuximab (0.1 mg/ml) or control Ab and HLA-G-blocking antibody (Cat. No. MAB11223, Abnova, 10 μg/ml)/KIR2DL4-blocking antibody (Cat. No. MAB10178, Abnova, 20 μg/ml)/ILT2-blocking antibody (Cat. No. 333721, BioLegend, 10 μg/ml)/PD-1-blocking antibody (Cat. No. 329902, BioLegend, 10 μg/ml)/PD-L1-blocking antibody (Cat. No. 329702, BioLegend, 10 μg/ml)/IL-12- blocking antibody (Cat. No. ab25036, Abcam, 10 μg/ml) were added before the ADCC assay. After 4 h, lysed target cells were labeled with propidium iodide (PI) at a final concentration of 50 mg/ml. Antibody-mediated cytotoxicity was analyzed by FCM. Lysis was quantified by calculating the percentage of PI-positive cells in all CFSE-positive target cells. Specific cell lysis was calculated using the following equation: % of specific lysis = (% of lysis of sample − % of lysis of control)/(100% − % of lysis of control).

### NK cell degranulation assay

Degranulation of NK cells was measured as previously described.^24^ Briefly, activated NK cells were mixed with target cells at an effector-to-target ratio of 1:1 in the presence of FITC-labeled anti-CD107a antibody (BioLegend) and incubated for 1 or 2 h at 37 °C. Where indicated, target cells were first incubated with trastuzumab (0.105 mg/ml) before coculture with NK cells. Surface expression of CD107a was measured using a FACS scan flow cytometer (BD Biosciences), and data were analyzed using the FlowJo software program.

### Histochemical analysis

Paraffin-embedded tissue sections were subjected to IHC staining as described previously with minor modifications.^47^ Briefly, the slides were deparaffinized in xylene and rehydrated in a graded alcohol series, and the endogenous peroxidase activity was blocked with 3% H_2_O_2_. Nonspecific binding sites were also blocked using preimmune rabbit serum before incubation of the sections overnight in a humidity chamber at 4 °C with the following primary antibodies: anti-CD56 (Cat. No. MAB24081, R&D Systems, Minneapolis, USA), anti-HLA-G (Cat. No. ab52455, Abcam), anti-KIR2DL4 (Cat. No. ab154386, Abcam), anti-PD-L1 (Cat. No. ab174838, Abcam), and anti-PD-1 (Cat. No. ab137132, Abcam). Slides were then washed three times with PBS, followed by incubation with a biotinylated secondary antibody for 30 min at room temperature. Signal was visualized by incubation with 3,3′-diaminobenzidine chromogen for 2–3 min. The IHC results were quantified according to the staining intensity and extent as previously described.^47^ For immunofluorescent staining of tumor tissues, the sections were deparaffinized and blocked as aforementioned, and were incubated at 4 °C overnight with primary antibodies against HER2 (Cat. No. ab11710, Abcam) and KIR2DL4 (Cat. No. ab154386, Abcam). Subsequently, incubation with goat anti-rat IgG H&L (Alexa Fluor® 488) (Cat. No. ab150157, Abcam) and goat anti-rabbit IgG H&L (Alexa Fluor® 594) (Cat. No. ab150080, Abcam) was performed for 1 h at 37 °C in the dark. Nuclei were counterstained with 4′,6-diamidino-2-phenylindole (Beyotime). Fluorescent images were acquired by a confocal laser-scanning microscope (T*i*2-E-A1, Nikon, Tokyo, Japan).

### Analysis of tumor growth

The animal study protocol was approved by the Ethics Committee of the Fourth Military Medical University (Permit Number: KY20163297-1). BALB/c nude mice (6 weeks old, 18 ± 0.39 g weight, female) were assigned randomly to each group for different analyses. Five million luciferase-expressing SK-BR-3 tumor cells or GKO #3 SK-BR-3 cells with matrix were injected subcutaneously into the right back near the thigh of the nude mice. When tumor volume reached 200 mm^3^ (defined as day 0), the mice received intraperitoneal treatment with 1 × 10^7^ primary NK cells, trastuzumab (5 mg/kg), or KIR2DL4/HLA-G-blocking antibody (1 mg/kg, Cat. No. MAB10178 and MAB11223, Abnova) alone or combined with NK cells and antibodies on days 0, 3, 6, 9, and 12. Tumor diameter was measured using a Vernier caliper every 5 days from day 5 until day 30. Tumor size was estimated using the formula: tumor volume = length × width^2^/2, where the length and width represented the longest and shortest tumor diameters. For bioluminescent imaging of in vivo tumors, the mice were injected intraperitoneally with 150 mg/kg d-luciferin (Gold Biotechnology, St. Louis, USA) and images were acquired 10 min later using a Xenogen IVIS^®^ Lumina II Imaging System and Living Image 4.3.1 software (Caliper Life Sciences, Hopkinton, USA). At the end of the experiment, tumors were excised from euthanized mice for further analysis.

### Statistics

Statistical analyses were performed using SPSS Statistics version 18.0 (IBM). Data were graphically depicted using GraphPad Prism 6.0 (GraphPad Software). Error bars indicate the standard deviation of the mean. Independent experiments are presented individually or combined, as explained in the figure legends. Unpaired *t* tests were used to compare continuous variables unless indicated otherwise. Statistical significance was tested using a two-tailed unpaired or paired Student’s *t* test. Correlations were evaluated using Spearman’s rank correlation with linear regression. Proportions were compared using *χ*^2^ tests. Differences in survival curves constructed by the Kaplan–Meier method were assessed using log-rank test. All *P* values were based on a two-sided hypothesis. *P* values < 0.05 were considered significant.

## Supplementary information

Supplementary Figures and Tables

MATERIAL Clinical data for representative patients in trastuzumab response analysis

## Data Availability

All data supporting this paper are present within the paper and/or the Supplementary Materials. The original datasets are also available from the corresponding author upon request.
