# Supplementary material for: Interaction between HLA-G and NK cell receptor KIR2DL4 orchestrates HER2-positive breast cancer resistance to trastuzumab
Source: Signal Transduct Target Ther. 2021 Jun 23;6:236. doi: 10.1038/s41392-021-00629-w (PMC8219715; doi:10.1038/s41392-021-00629-w)
Supplement: Supplementary file 1 — Supplementary Figures and Tables [file 41392_2021_629_MOESM1_ESM.docx]

**Supplementary Materials for**

**“Interaction between HLA-G and NK cell receptor KIR2DL4 orchestrates HER2-positive breast cancer resistance to trastuzumab”**

Guoxu Zheng^1,#^, Zhangyan Guo^1,#^, Weimiao Li^2,#^, Wenjin Xi^1^, Baile Zuo^1^, Rui Zhang^1^, Weihong Wen^1^, An-Gang Yang^1,*^, Lintao Jia^3,*^

^1^State key laboratory of Cancer Biology, Department of Immunology, Fourth Military Medical University, Xi’an, 710032, China

^2^Department of Oncology, the Second Affiliated Hospital of Xi'an Jiaotong University, Xi'an, 710004, China

^3^State key laboratory of Cancer Biology, Department of Biochemistry and Molecular Biology, Fourth Military Medical University, Xi’an, 710032, China

^#^These authors contributed equally

**Correspondence to:** Lintao Jia ([jialth@fmmu.edu.cn](mailto:jialth@fmmu.edu.cn)) and An-Gang Yang ([agyang@fmmu.edu.cn](mailto:agyang@fmmu.edu.cn)); Phone: (86)-29-84712321; Fax: (86)-29-84773947

**Conflicts of interests:** The authors declare no competing interests.

**This file includes:**

Figures S1 to S13

Tables S1 to S2

**Supplementary Tables:**

**Table S1. The association of HLA-G expression with clinical characteristics of patients with HER2-positive breast cancer**

| **Clinical characteristics** | **Case number**  **(n=108)** | **HLA-G expression** | | | | ***P*** |
| --- | --- | --- | --- | --- | --- | --- |
|  |  | **Positive (+, ++, +++)**  **(n=71)** | | | **Negative (-)**  **(n=37)** |  |
| **Age(years)** |  |  | | | | 0.347 |
| **≥60** | 56 | 34 | 22 | | |  |
| **<60** | 52 | 37 | 15 | | |  |
| **Gender** |  |  |  | | | 0.738 |
| **Male** | 1 | 0 | 1 | | |  |
| **Female** | 107 | 71 | 36 | | |  |
| **Tumor size** |  |  | | | | 0.605 |
| **≥3** | 46 | 32 | | 14 | |  |
| **<3** | 62 | 39 | | 23 | |  |
| **Tumor differentiation** |  |  | | | | 0.004* |
| **Poorly/moderately** | 73 | 41 | | 32 | |  |
| **Well** | 35 | 30 | | 5 | |  |
| **TNM stage** |  |  | | | | 0.003* |
| **I-II** | 45 | 22 | | 23 | |  |
| **III-IV** | 63 | 49 | | 14 | |  |
| **Lymph node metastasis** |  |  | | | | 0.849 |
| **Yes** | 67 | 45 | | 22 | |  |
| **No** | 41 | 26 | | 15 | |  |

**P* <0.05

**Table S2. Cox regression analysis of prognostic factors for overall survival in HER2-positive breast cancer patients (n=108)**

|  | **Univariate analysis** | | **Multivariate analysis** | |
| --- | --- | --- | --- | --- |
|  | *P* value | HR (95%CI) | *P* value | HR (95%CI) |
| **HLA-G expression** | 0.000* | 6.287 (3.158-18.527) | 0.000* | 7.356 (3.526-19.466) |
| **Age** | 0.280 | 1.011 (0.991-1.032) | 0.912 | 1.001 (0.980-1.023) |
| **Gender** | 0.389 | 0.773 (0.431-1.388) | 0.099 | 0.585 (0.314-1.104) |
| **Tumor size** | 0.062 | 0.382 (0.209-1.698) | 0.058 | 0.233 (0.117-1.465) |
| **TNM stage** | 0.042* | 2.685 (1.362-8.233) | 0.037* | 3.434 (2.243-9.867) |

95% CI indicates 95% confidence interval

**P* < 0.05

**Supplementary Figures:**


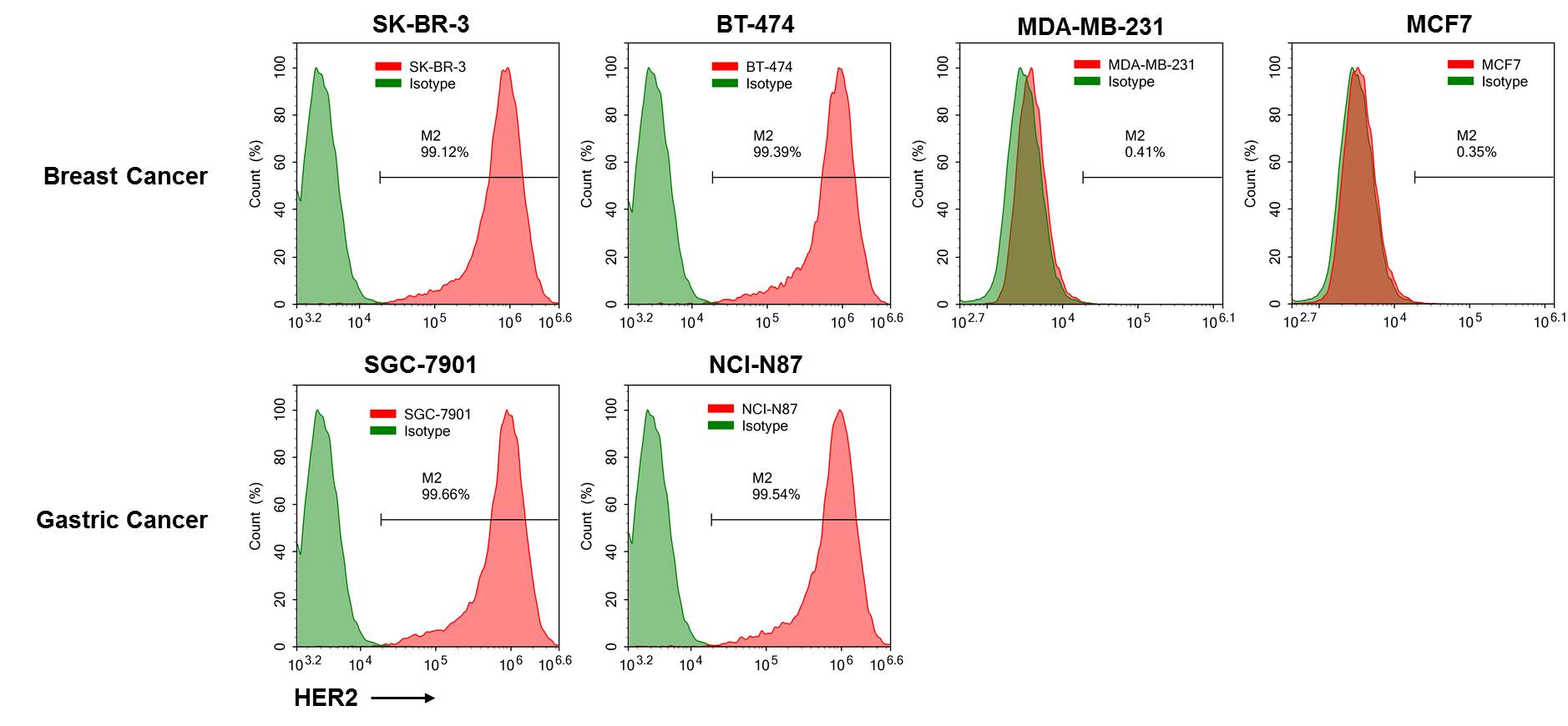


**Figure S1. FCM assay of HER2 expression on human breast cancer and gastric cancer cell lines.**


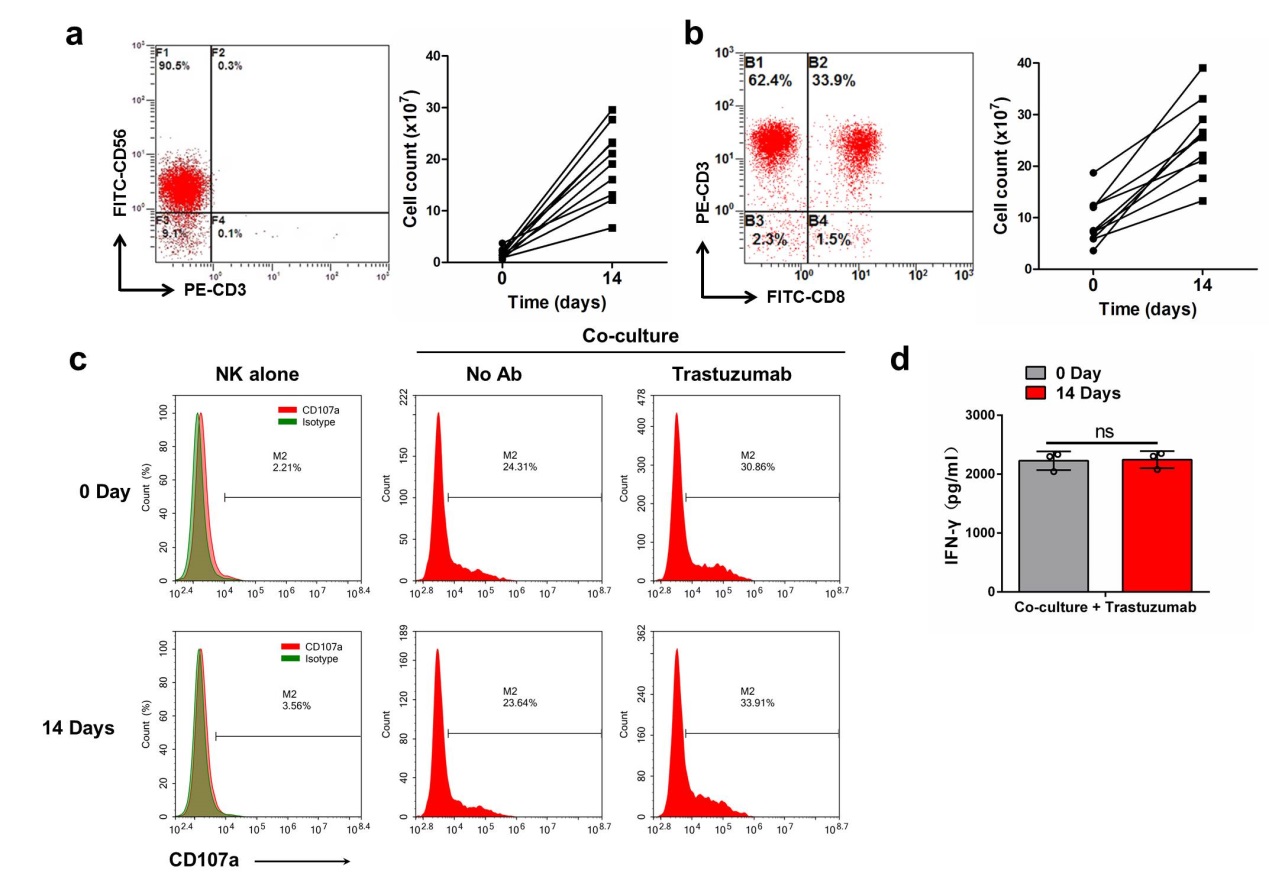


**Figure S2. Characteristics of NK and T cells isolated from human PBMCs and expanded *in vitro*.**

(a, b) NK cells (CD56^+^CD3^-^, a) and CD3+ T cells (b) were obtained from PBMC by magnetic bead separation and subjected to FCM analysis. Cells were counted before and after expanded culture for 2 weeks. (c, d) NK cells in (a) were cultured alone or co-cultured with SK-BR-3 cells in the presence or absence of trastuzumab, and were subjected to FCM analysis after staining for the degranulation marker, CD107a (c), or subjected to ELISA (d). ns, nonsignificant.





**Figure S3. Trastuzumab evokes lysis of SK-BR-3 cells by NK cells but not T cells.**

NK or T cells were co-cultured with SK-BR-3 cells for 4 hours in the presence of trastuzumab or an IgG control, followed by cytotoxicity assay as described in Materials and methods. All experiments were performed 3 times. Statistical significance was assessed by Student’s *t*-test. ***P* < 0.01. ns, non-significant.


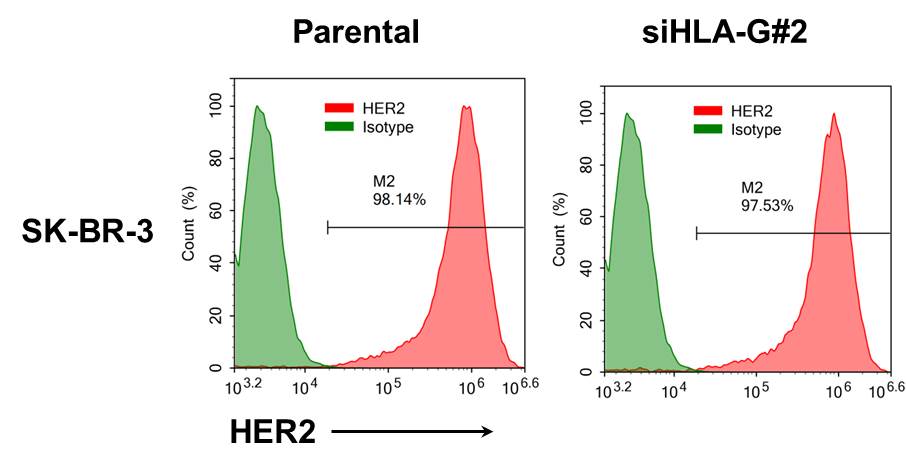


**Figure S4. HLA-G knockdown fails to affect HER2 expression on SK-BR-3 cells.**

Cells were unmodified (Parental) or transfected with HLA-G siRNA (siHLA-G#2), and were subjected to FCM analysis for HER2 expression.


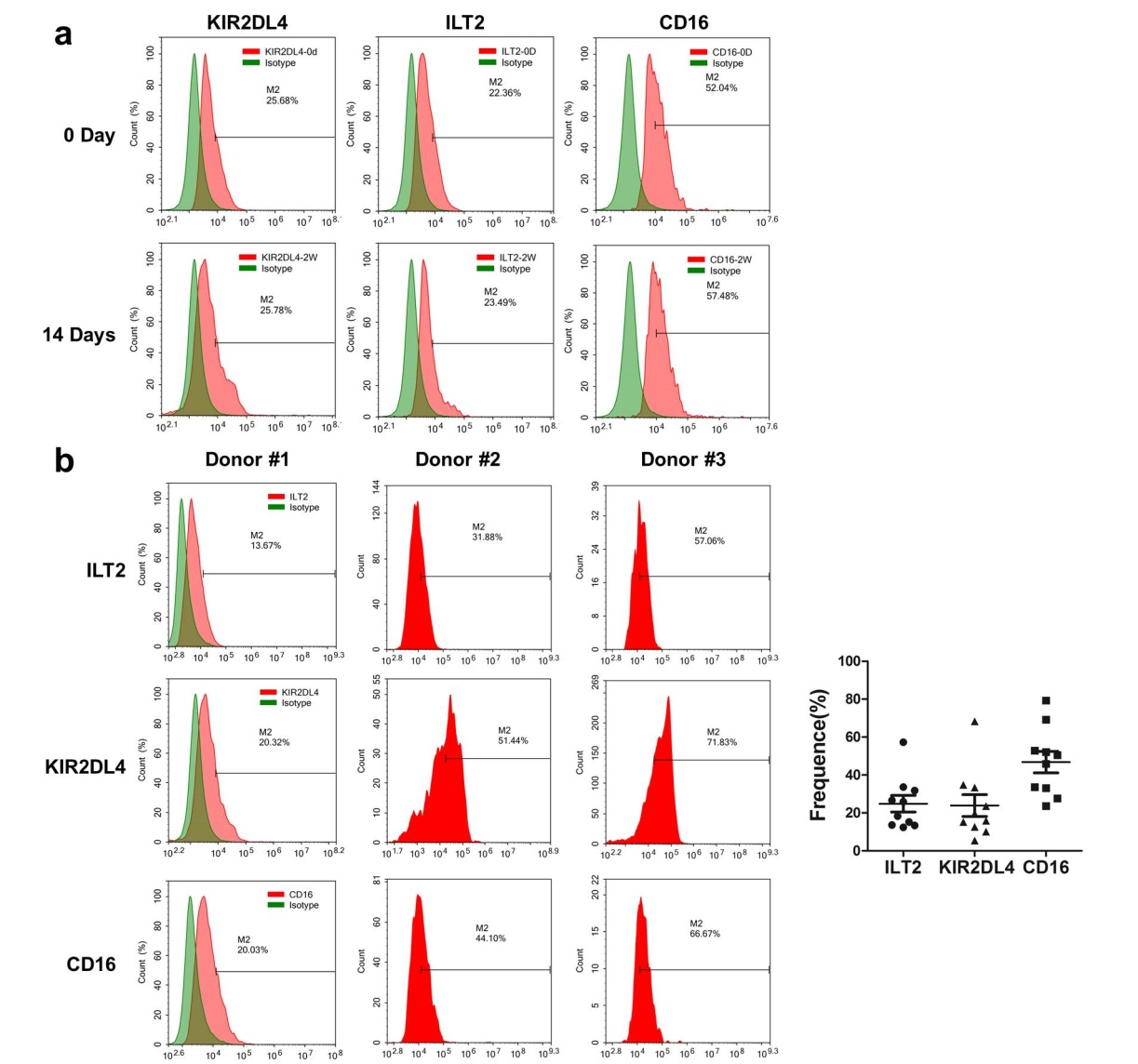


**Figure S5. FCM assays of ILT2, KIR2DL4 and CD16 expression on human NK cells.**

1. NK cells prepared from PBMCs were stained for surface receptors and subjected to FCM analysis on indicated days of the *in vitro* expansion protocol. (b) NK cells prepared from PBMCs of different donors were expanded and underwent FCM analysis.


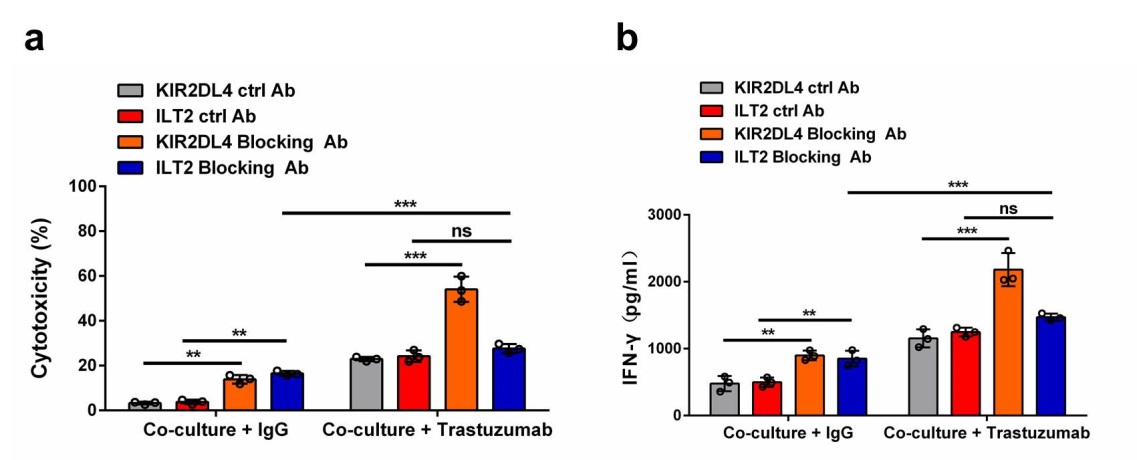


**Figure S6. Blocking KIR2DL4 but not ILT2 enhances trastuzumab-induced ADCC and IFN-γ production by NK cells.**

NK cells were cocultured with SK-BR-3 cells (E:T=30:1) in the absence or presence trastuzumab supplemented with the indicated blocking or control antibodies. The toxicity of NK cells to malignant cells was measured via FCM (a), and IFN-γ production was measured via ELISA (b). All experiments were performed 3 times. Statistical significance was determined by Student’s *t*-test. ***P* < 0.01, ****P* < 0.001. ns, nonsignificant.


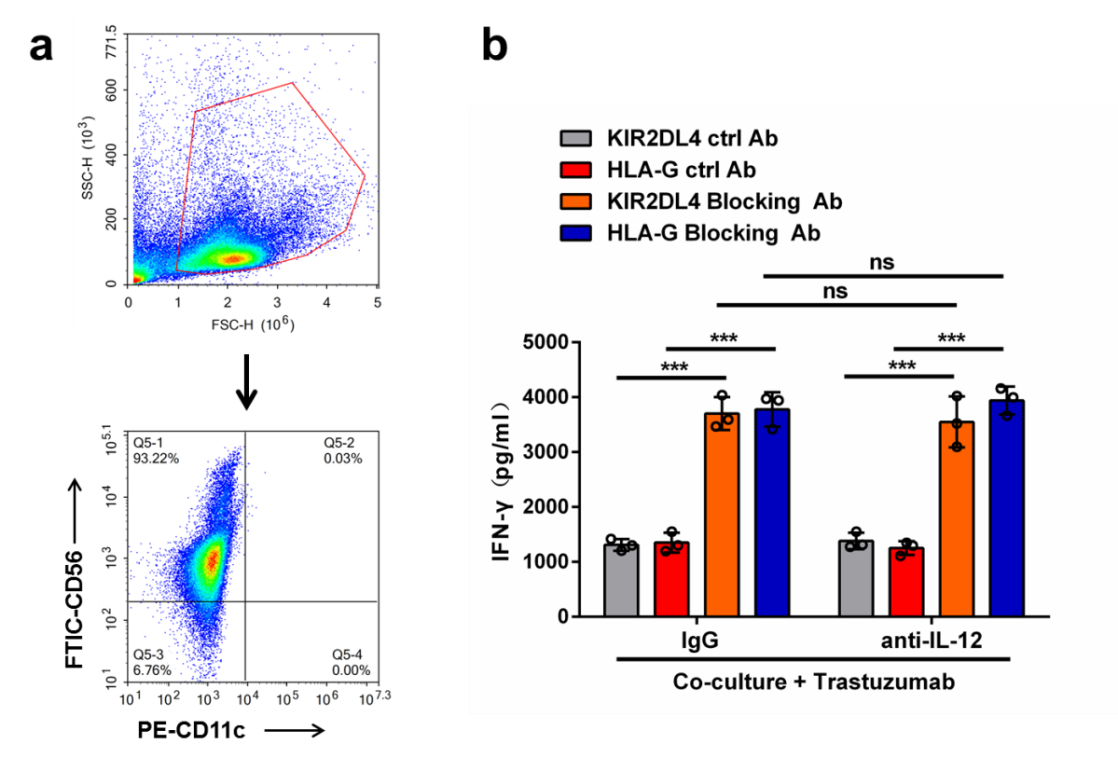


**Figure S7. IFN-γ production in cocultured breast cancer and NK cells was not due to contamination of DCs.**

(a) NK cells prepared from PBMCs of healthy donors were subjected to FCM for expression of the DC marker CD11c. (b) NK cells were cocultured with SK-BR-3 cells (E:T=30:1) in the presence trastuzumab supplemented with the indicated blocking or control antibodies. The production of IFN-γ was measured via ELISA. All experiments were performed 3 times. Statistical significance was determined by Student’s *t*-test. ***P* < 0.01, ****P* < 0.001. ns, nonsignificant.


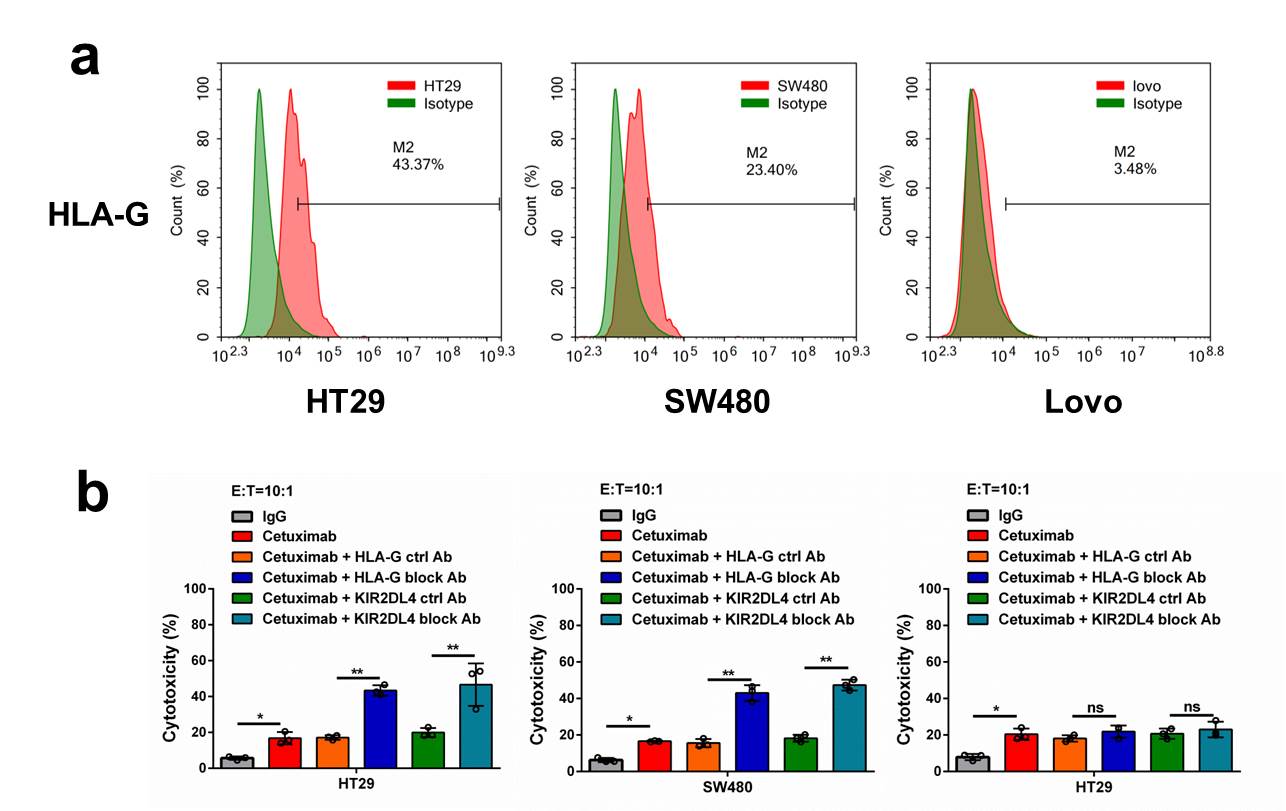


**Figure S8. HLA-G blockade improves cetuximab-mediated killing of colorectal cancer cells by co-cultured NK cells.**

(a) FCM assay for HLA-G expression on colorectal cancer cell lines. (b) NK cells were co-cultured with colorectal cancer cells in the presence of cetuximab alone or together with the indicated antibodies, followed by cytotoxicity assay via FCM. **P* < 0.05, ***P* < 0.01. ns, nonsignificant.


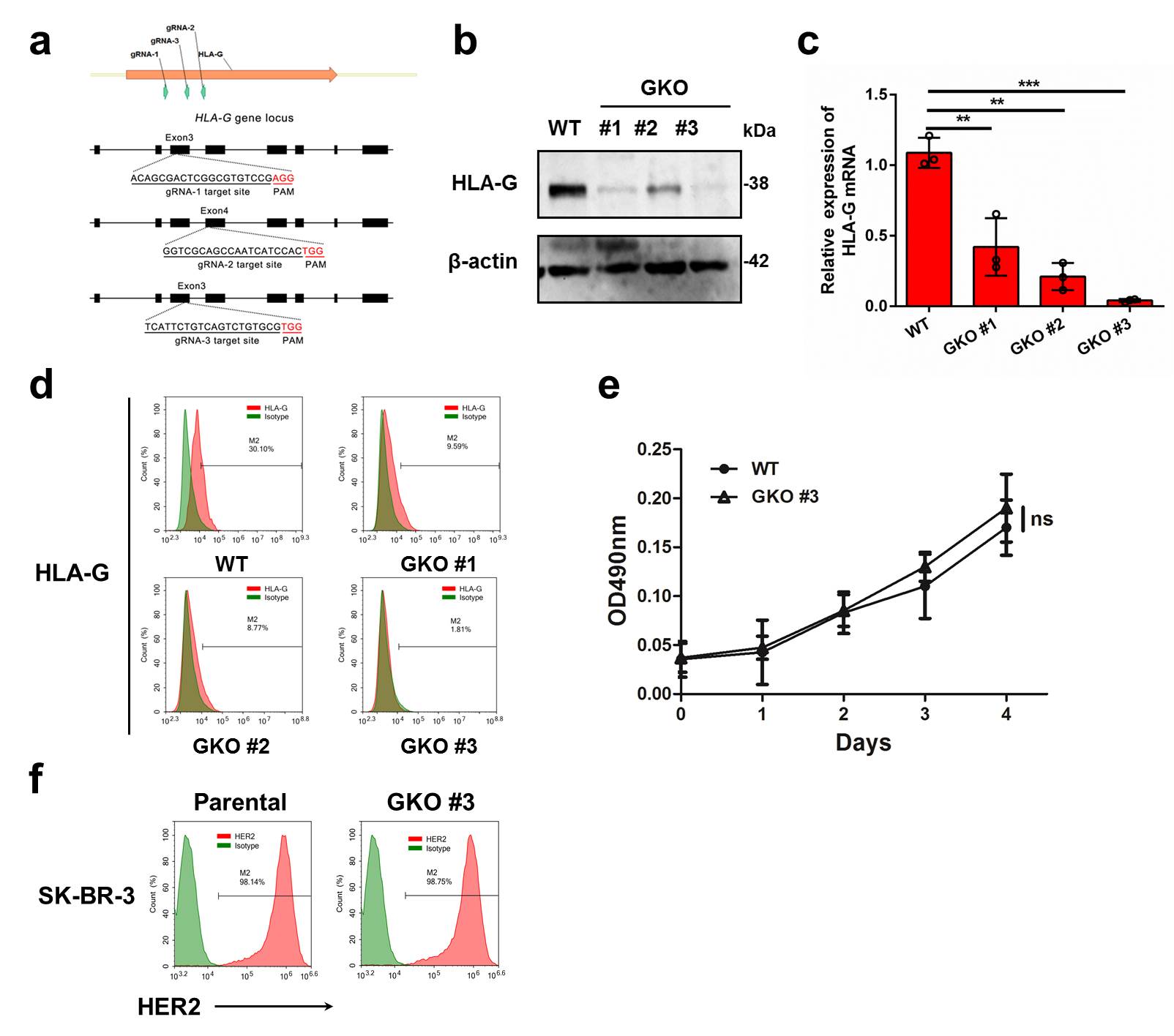


**Figure S9. Generation of *HLA-G* knockout SK-BR-3 cells via CRISPR/Cas9.**

(a) Schematic diagram showing the genomic locus and the sgRNA targeting sites for the HLA-G (NM_002127) gene. (b-d) Western blot (b), qRT-PCR (c) and FCM assays (d) for HLA-G expression in wild type (WT) and gene knockout clones (GKO #1, #2, #3) of SK-BR-3 cells. (e) The proliferation of WT and GKO #3 SK-BR-3 cells were measured by MTT assay. (f) Parental and GKO #3 SK-BR-3 cells were subjected to FCM assay for HER2 expression. All experiments were performed 3 times. Statistical significance was assessed by Student’s *t* test. ***P* < 0.01, ****P* < 0.001. ns, nonsignificant.


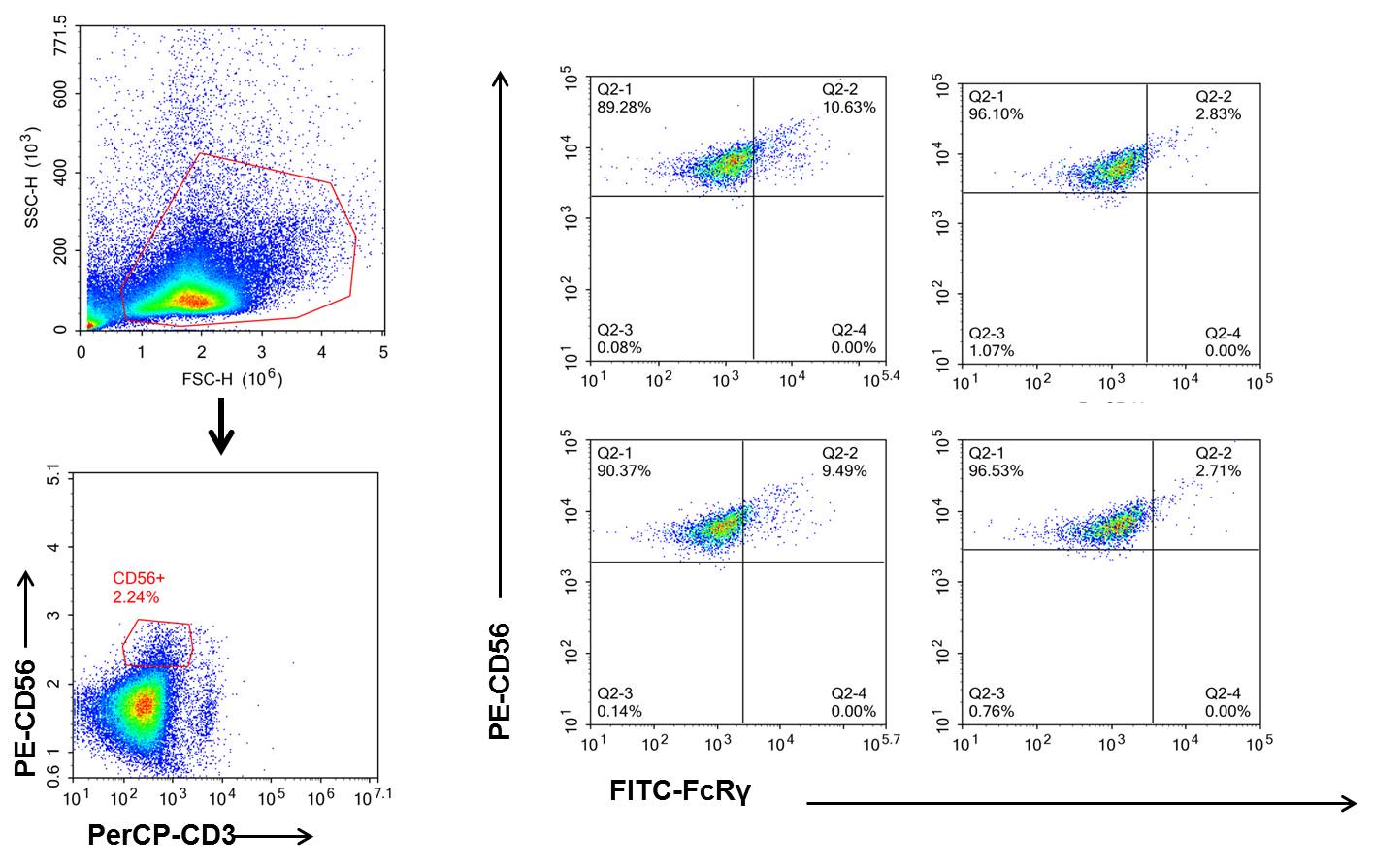


**Figure S10. FcRγ is expressed in tumor-infiltrating NK cells in clinical HER2-positive breast cancer.**

Fresh tumors were obtained from HER2-positive breast cancer patients undergoing surgery. Cells were disassociated and subjected to FCM for NK cells (CD56^+^CD3^-^) as described in Materials and Methods, and the representative results were shown (*left panel*). The gated CD56^+^CD3^-^ cells were then analyzed via FCM for FcRγ expression, and the results for tumors from different individual patients were shown (*right panel*).


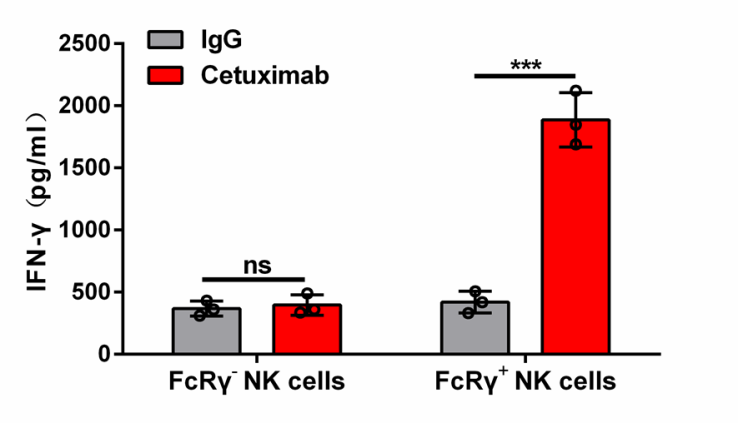


**Figure S11. Cetuximab induces IFN-γ secretion by NK cells cocultured with EGFR-positive colon cancer HT29 cells.**

FcRγ^+^ or FcRγ^-^ NK cells prepared from healthy donors were cocultured with human colon cancer HT29 cells in the presence of HLA-G-blocking antibody together with Cetuximab or an IgG control. The production of IFN-γ was then measured via ELISA. All experiments were performed 3 times. Statistical significance was determined by Student’s *t*-test. ****P* < 0.001. ns, nonsignificant.

**
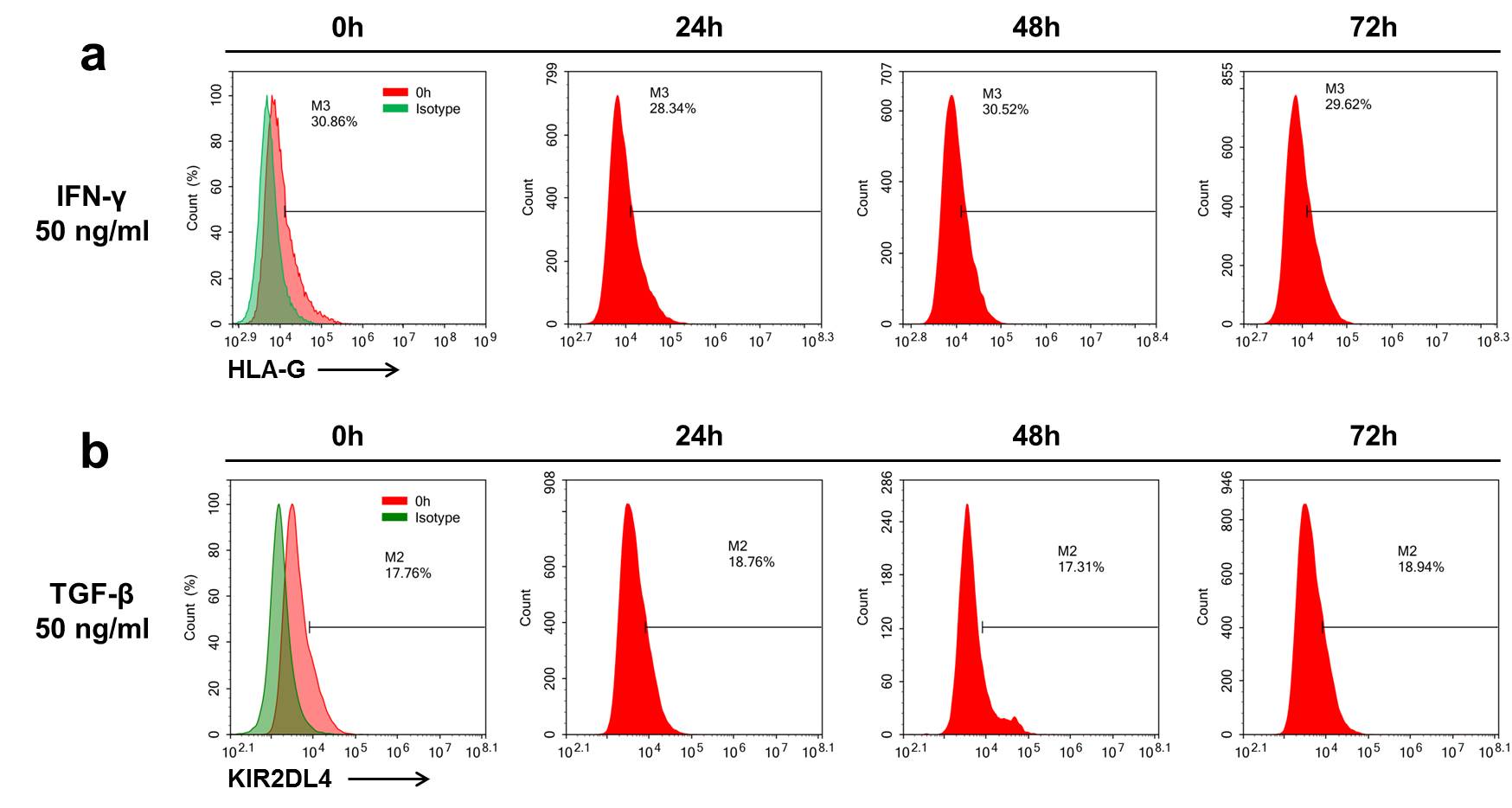
**

**Figure S12. IFN-γ does not affect HLA-G expression on breast cancer cells nor does TGF-β regulate KIR2DL4 on NK cells.**

(a) SK-BR-3 cells were incubated with IFN-γ for the indicated times and were subjected to FCM assay for determining HLA-G level. (b) NK cells were incubated with TGF-β for the indicated times and were subjected to FCM assay for the expression of KIR2DL4.

**
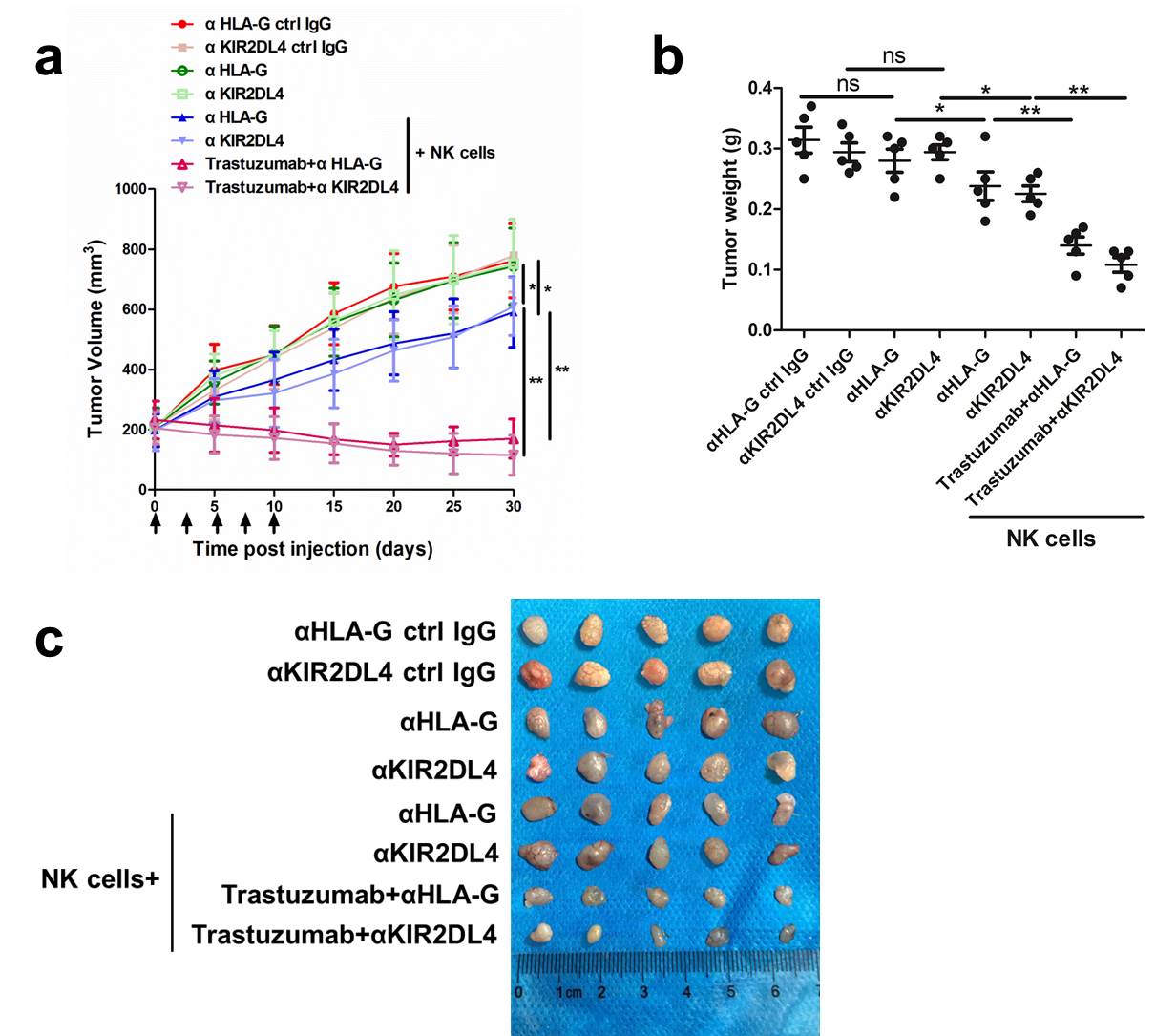
**

**Figure S13. Blockade of HLA-G/KIR2DL4 signaling facilitates tumor suppression via enhanced ADCC.**

(a-c) Nude mice were inoculated with SK-BR-3 cells, followed by intraperitoneal treatment with 1×10^7^ primary NK cells, trastuzumab (5 mg/kg) and HLA-G/KIR2DL4 blocking antibody (1 mg/kg) on days 0, 3, 6, 9 and 12. Tumor volumes of mice were monitored and plotted (a). Mice were sacrificed on day 30, and tumors were excised and weighed (b, c). All experiments were performed 3 times. Statistical significance was assessed by Student’s *t* test. **P* < 0.05, ***P* < 0.01. ns, nonsignificant.
