# Supplementary material for: Interaction between HLA-G and NK cell receptor KIR2DL4 orchestrates HER2-positive breast cancer resistance to trastuzumab
Source: Signal Transduct Target Ther. 2021 Jun 23;6:236. doi: 10.1038/s41392-021-00629-w (PMC8219715; doi:10.1038/s41392-021-00629-w)
Supplement: Supplementary file 2 — MATERIAL Clinical data for representative patients in trastuzumab response analysis [file 41392_2021_629_MOESM2_ESM.pdf]

Representative pathological reports and computerized tomography (CT) scan results for patients involved in trastuzumab sensitivity study

Resistant patient #3

Pathological report

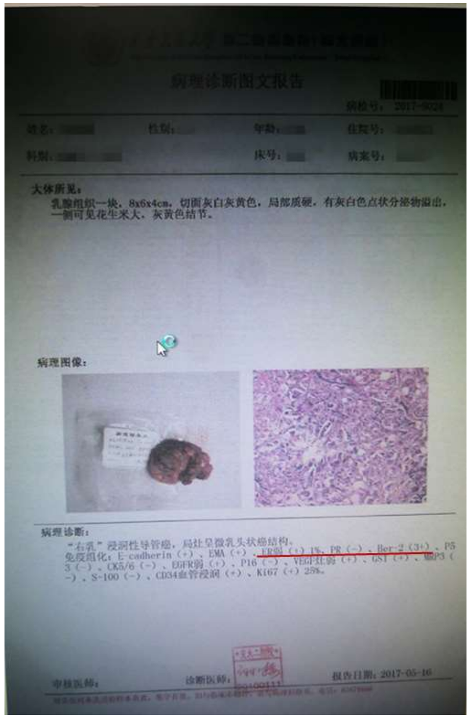

Pre-operative CT

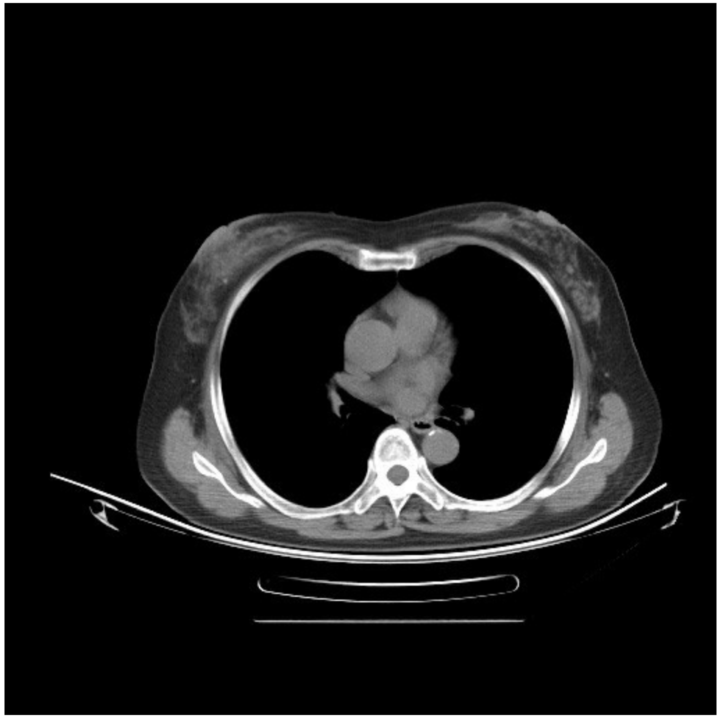

Post-operative CT

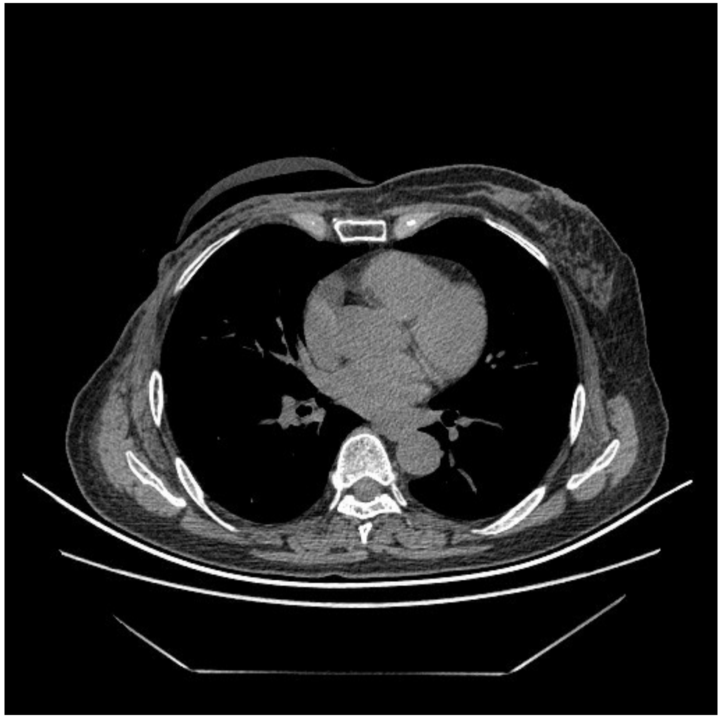

Pathologic Findings:

A piece of mammary gland tissue (8x6x4 cm), with grayish yellow cut surface, local hard tumor texture, overflow of grayish punctate secretion, grayish yellow nodules as large as a peanut on one side.

Pathologic Diagnosis:

Ductal Infiltrating carcinoma of the right breast with micropapillary carcinoma architecture in local foci.

Immunohistochemical results:

E-cadherin (+), EMA(+), ER weak(+) 1%, PR(-), Her-2(3+), P53(-), CK5/6(-), EGFR weak(+), P16(-), VGFR weak(+), GST(+), MRP3 (-), S-100 (-), vascular infiltrating CD34(+), Ki67 (+) 25%

Resistant patient #12

Pathological report

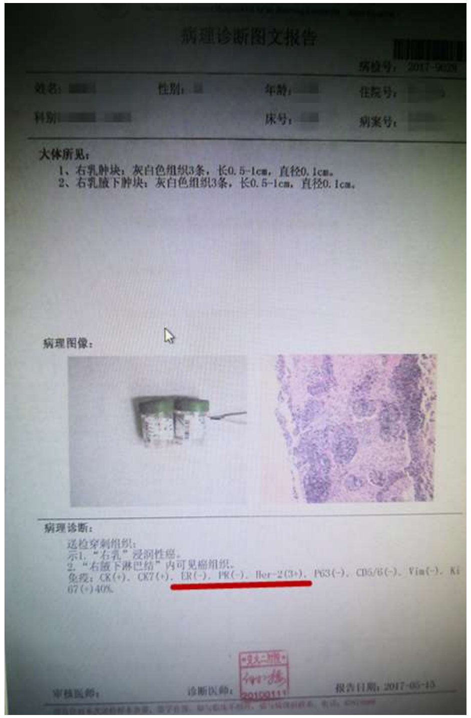

病理诊断图文报告

病检号：2017-90[illegible]

姓名：[illegible] 性别：[illegible] 年龄：[illegible] 住院号：[illegible]

科别：[illegible] 床号：[illegible] 病案号：[illegible]

大体所见：

1、右乳肿块：灰白色组织3条，长0.5-1cm，直径0.1cm。
2、右乳腋下肿块：灰白色组织3条，长0.5-1cm，直径0.1cm。

病理图像：

病理诊断：

送检穿刺组织：
示1."右乳"浸润性癌。
2."右腋下淋巴结"内可见癌组织。
免疫：CK(+)、CK7(+)、ER(-)、PR(-)、Her-2(3+)、P63(-)、CD5/6(-)、Vim(-)、Ki67(+)40%。

审核医师： 诊断医师： 报告日期：2017-05-15

Pre-operative CT

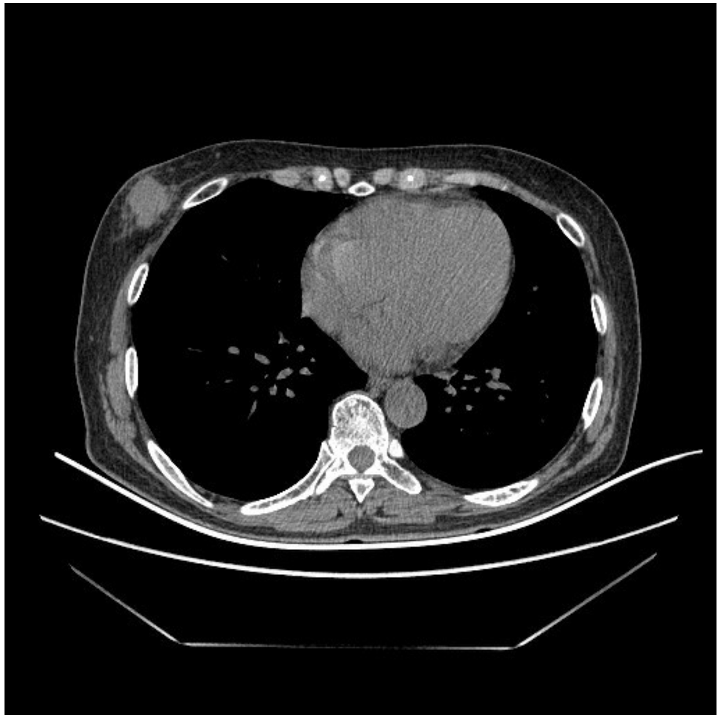

Post-operative CT

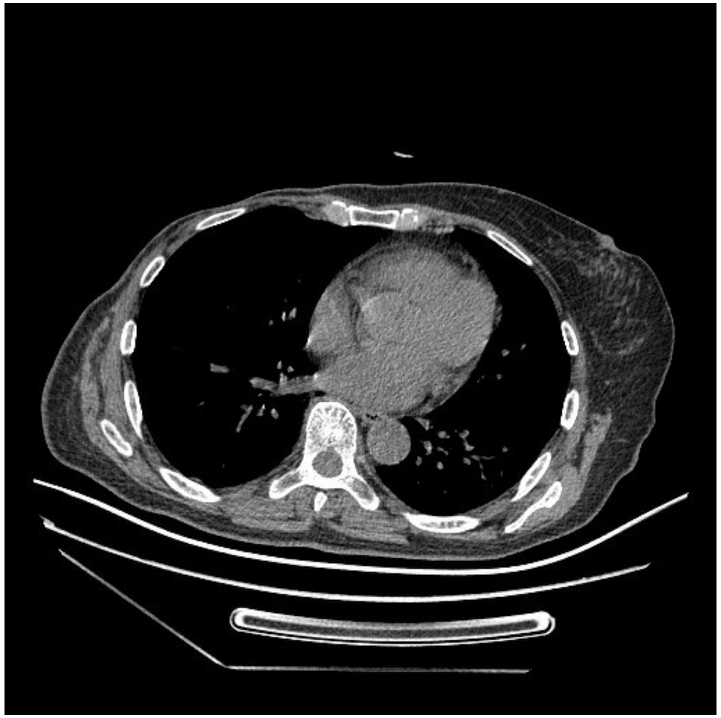

Pathologic Findings:

1. Right breast mass, 3 strips of grayish white tissue, 0.5-1 cm in length and 0.1 cm in diameter.
2. Axillary mass in the right breast, 3 strips of grayish white tissues, 0.5-1 cm in length and 0.1 cm in diameter

Pathologic Diagnosis:

Submitted puncture tissue;

1. Infiltrating carcinoma of the right breast.
2. Cancer tissue was observed in the right axillary lymph node.

Immunohistochemical results:

CK(+), CK7(+), ER (-), PR(-), Her-2(3+), P63(-), CD5/6(-), Vim(-), Ki67 (+) 40%

Sensitive patient #19

Pathological report

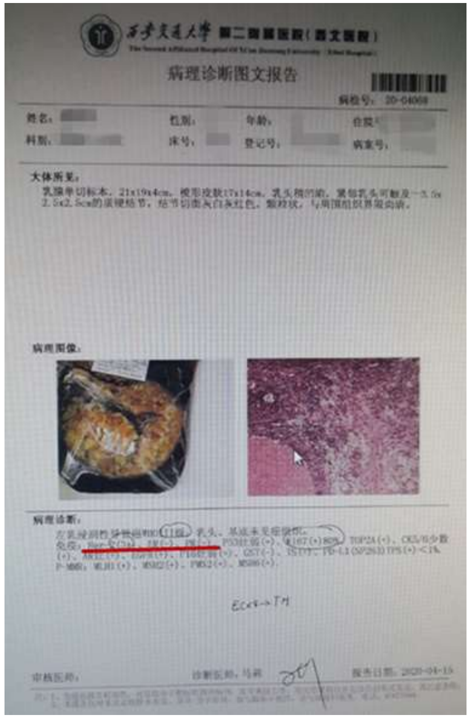

Pre-operative CT

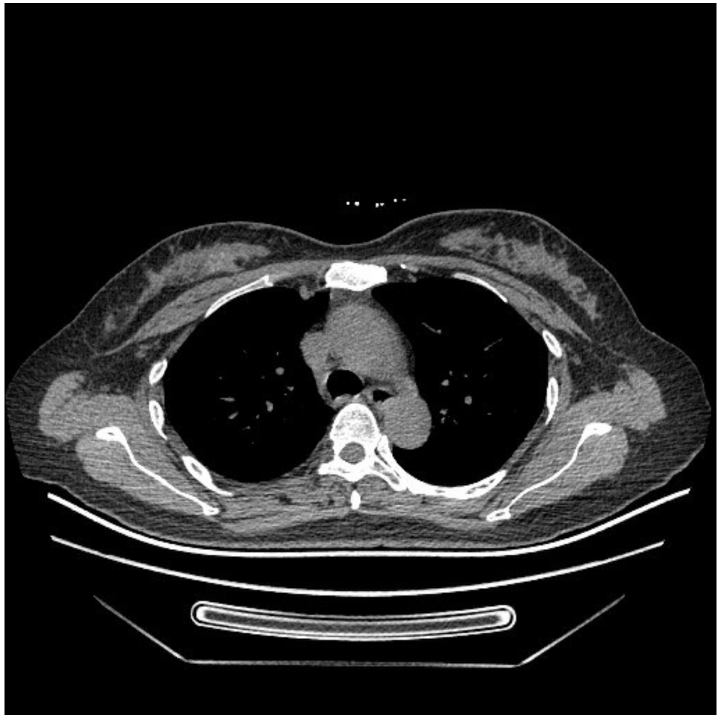

Post-operative CT

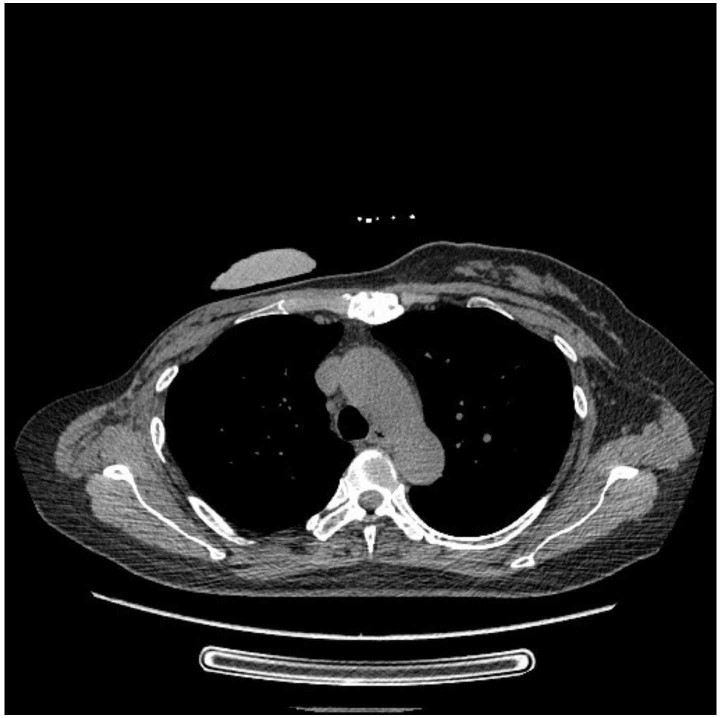

Pathologic Findings:

Single mastectomy breast specimens (21x19x4 cm), shuttle type skin (17x14 cm), slightly depressed papillae, palpable hard nodule adjacent to papillae (3.5x2.5x2.5 cm), with grayish red cut surface, granular; the boundary with the surrounding tissues was still clear.

Pathologic Diagnosis:

Ductal Infiltrating carcinoma of the left breast (NOS grade II); no cancer tissue was observed in the base tissue.

Immunohistochemical results:

Her-2(3+), ER(-), PR(-), P53weak(+), Ki67 (+) 80%, TOP2A(+), CK5/6weak(+), AR(+), EGFR (+), P16weak(+), GST(-), TS(+), PD-L1(SP263)TPS(+) <1%

MMR detection： MLH1(+), MSH2(+), PMS2(+), MSH6(+)

Sensitive patient #28

Pathological report

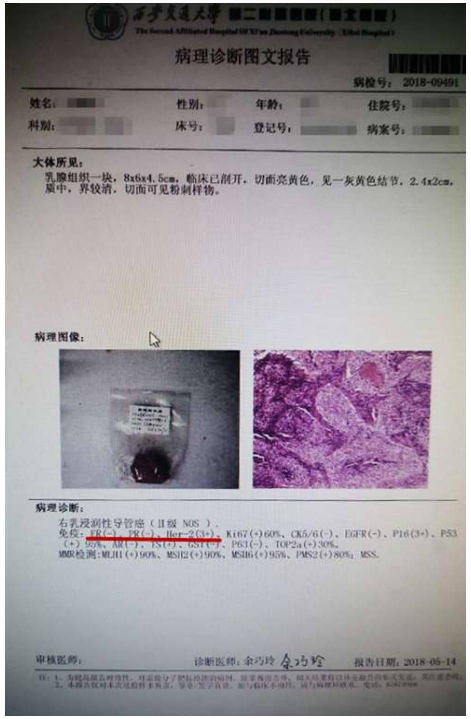

病理诊断图文报告

病检号：2018-09491

姓名：　性别：　年龄：　住院号：

科别：　床号：　登记号：　病案号：

大体所见：

乳腺组织一块，8x6x4.5cm，临床已剖开，切面亮黄色，见一灰黄色结节，2.4x2cm，质中，界较清，切面可见粉刺样物。

病理图像：

病理诊断：

右乳浸润性导管癌（II级 NOS）。

免疫：ER(-)、PR(-)、Her-2(3+)、Ki67(+)60%、CK5/6(-)、EGFR(-)、P16(3+)、P53(+)95%、AR(-)、TS(+)、GST(-)、P63(-)、TOP2a(+)30%。

MMR检测：MLH1(+)90%、MSH2(+)90%、MSH6(+)95%、PMS2(+)80%；MSS。

审核医师：　诊断医师：余巧玲　报告日期：2018-05-14

Pre-operative CT

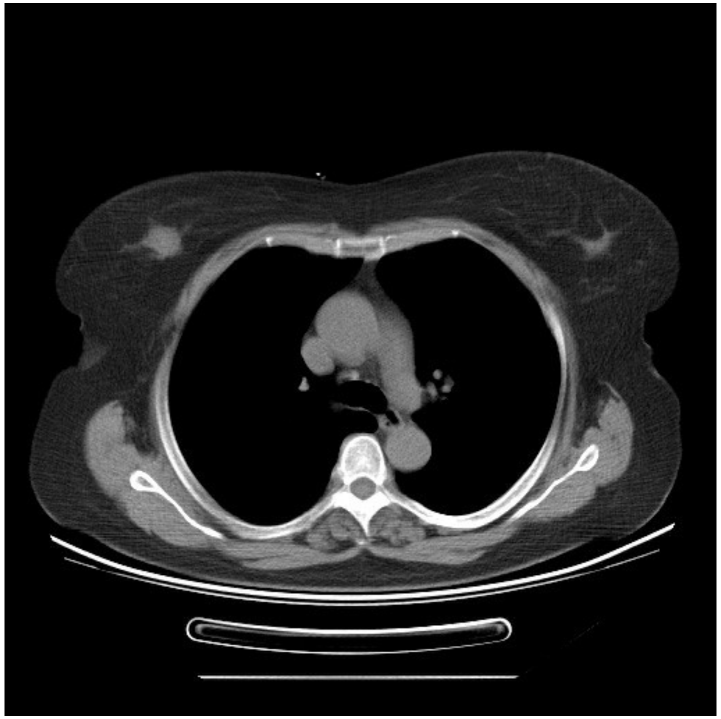

Post-operative CT

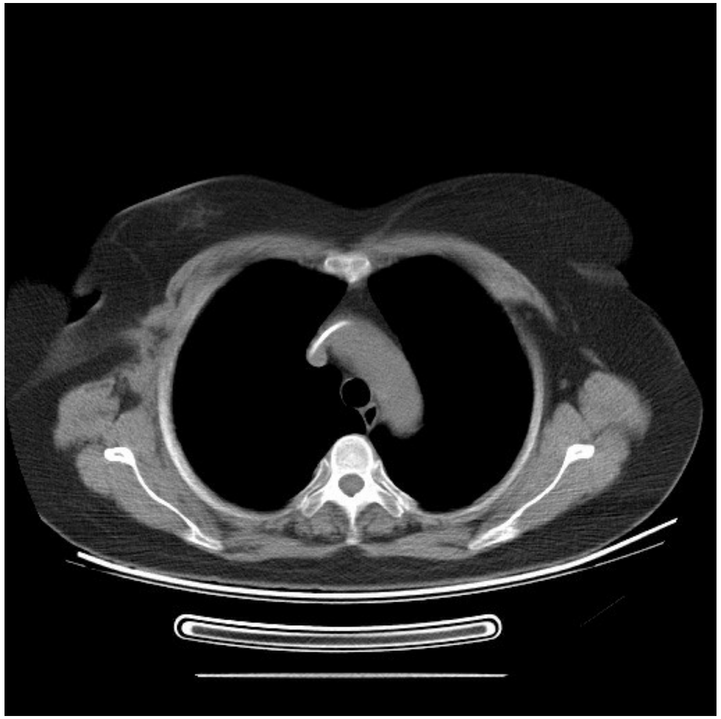

Pathologic Findings:

A piece of mammary gland tissue (8x6x4.5 cm), clinically dissected, with bright yellow cut surface, a grayish yellow nodules (2.4x2 cm) found, medium tumor texture, clear boundary, visible comedo sample on cut surface.

Pathologic Diagnosis:

Ductal Infiltrating carcinoma of the right breast (NOS grade II)

Immunohistochemical results:

ER(-), PR(-), Her-2(3+), Ki67 (+) 60%, CK5/6(-), EGFR (-), P16(3+), P53(+) 95%, AR(-), TS(+), GST(-), P63(-), TOP2a(+)30%

MMR detection： MLH1(+) 90%, MSH2(+) 90%, MSH6(+) 95%, PMS2(+) 80%, MSS
